# Clinical correlates of lipoprotein (a) and apolipoprotein B levels in patients with dyslipidemia and cardiovascular disease

**DOI:** 10.3389/fcvm.2026.1821490

**Published:** 2026-07-09

**Authors:** Luana Alexandrescu, Daria Maria Alexandrescu, Ionut Tiberiu Tofolean, Doina Ecaterina Tofolean, Steliana Pindichi, Eugen Dumitru, Bogdan Campineanu, Cristina Aftenie, Andreea Nelson Twakor, Alexandra Herlo, Elena Rusu, Diana Raluca Baicu, Madalina Ilie, Filip-Vasile Berariu, Laura Maria Condur

**Affiliations:** 1 Gastroenterology Department, “Sf. Apostol Andrei” Emergency County Hospital, Constanta, Romania; 2 Medicine Faculty, “Ovidius” University of Constanta, Constanta, Romania; 3 Faculty of Medicine, Titu Maiorescu University, Bucharest, Romania; 4 Pneumology Department, Sf. Apostol Andrei” Emergency County Hospital, Constanta, Romania; 5 Doctoral School, Medicine Faculty, “Ovidius” University of Constanta, Constanta, Romania; 6 Nephrology Department, “Sf. Apostol Andrei” Emergency County Hospital, Constanta, Romania; 7 Internal Medicine Department, Sf. Apostol Andrei” Emergency County Hospital, Constanta, Romania; 8 Department XIII, Discipline of Infectious Diseases, “Victor Babes” University of Medicine and Pharmacy Timisoara, Timisoara, Romania; 9 Doctoral School, University of Medicine and Pharmacy “Carol Davila” Bulevardul Eroii Sanitari 8, Bucharest, Romania; 10 Department of Gastroenterology, University of Medicine and Pharmacy “Carol Davila” Bulevardul Eroii Sanitari 8, Bucharest, Romania; 11 Plastic and Reconstructive Surgery, Regional Institute of Oncology, Iasi, Romania

**Keywords:** apolipoprotein B, cardiovascular disease, creatinine, dyslipidemia, gamma-glutamyl transferase, lipoprotein(a), uric acid

## Abstract

**Background:**

Atherosclerotic cardiovascular disease remains a major cause of morbidity and mortality worldwide, with residual cardiovascular risk persisting despite optimized lipid-lowering therapy. Lipoprotein(a) [Lp(a)] and apolipoprotein B (ApoB) have emerged as independent, causal biomarkers of cardiovascular risk, reflecting atherogenic particle burden beyond conventional lipid measures. This study aimed to evaluate the clinical, metabolic, and biochemical correlates of Lp(a) and ApoB levels in patients with dyslipidemia and cardiovascular disease.

**Methods:**

A cross-sectional observational analysis was performed in 153 adults evaluated for cardiometabolic risk. Anthropometric, biochemical, and inflammatory parameters were assessed. Correlation analyses were conducted to identify associations between lipoproteins and metabolic variables. Decision tree regression was used to explore distribution patterns of Lp(a) and ApoB variability.

**Results:**

The study population exhibited a mean age of 57.9 ± 12.3 years, with a predominance of dyslipidemia (79.1%) and established cardiovascular disease (75.2%). The mean Lp(a) concentration was 30.14 ± 31.50 mg/dL, while ApoB averaged 119.87 ± 36.01 mg/dL. ApoB correlated significantly with total cholesterol (*r* = 0.49, *p* < 0.001), triglycerides (*r* = 0.46, *p* < 0.001), and LDL-C (*r* = 0.26, *p* < 0.01). Decision tree analysis identified GGT, creatinine, and uric acid as factors that influence of Lp(a) variability.

**Conclusions:**

Both hepatic and renal function markers significantly modulate Lp(a) levels, while ApoB remains a robust indicator of atherogenic particle load. Integrating Lp(a) and ApoB assessment into cardiovascular risk profiling may improve identification of residual risk and guide personalized therapeutic strategies in dyslipidemic populations.

## 1 Introduction

**Figure 1:**
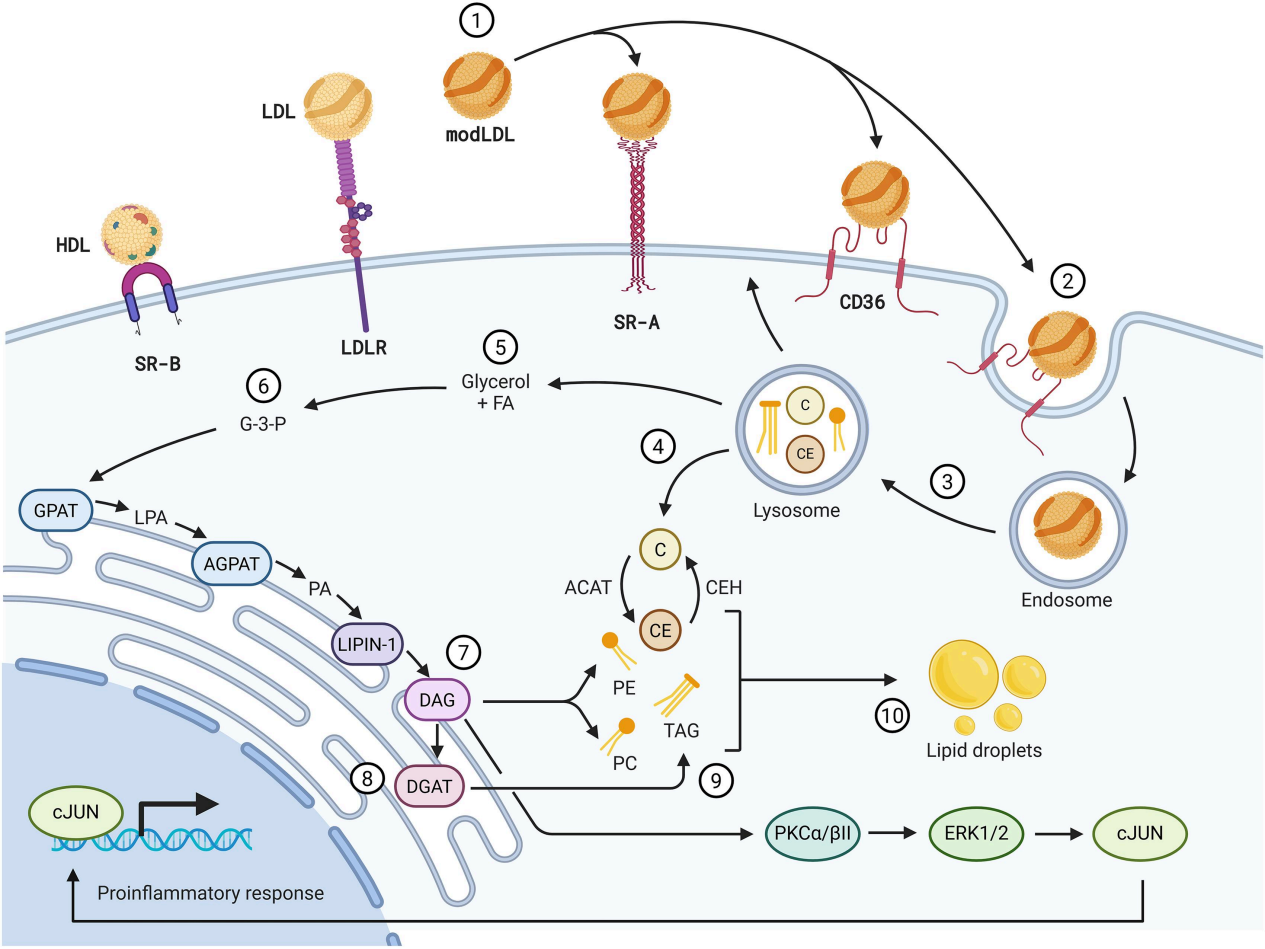
Cellular pathways of modified LDL uptake and intracellular lipid metabolism leading to proinflammatory activation. Modified LDL particles are recognized by scavenger receptors SR-A and CD36 (1–2), internalized into endosomes (3), and trafficked to lysosomes (4), where cholesteryl esters (CE) are hydrolyzed to free cholesterol (C). Re-esterification via ACAT and CEH regulates cholesterol balance and promotes lipid droplet formation (10). Concurrently, lipogenic enzymes including GPAT, AGPAT, and DGAT (6–8) drive the synthesis of triacylglycerol (TAG) and di-acylglycerol (DAG), activating PKCα/βII and ERK1/2 signaling (9–10). This cascade culminates in c-JUN–mediated transcriptional activation and a proinflammatory cellular phenotype.

Atherosclerotic cardiovascular disease (ASCVD) persists as a leading cause of morbidity and mortality globally, with lipid metabolism playing a pivotal role in its pathogenesis. Among the lipid parameters, Lp(a) and ApoB have emerged as independent, causal biomarkers of cardiovascular risk that extend beyond traditional lipid measures such as low-density lipoprotein cholesterol (LDL-C) (1). Elevated levels of Lp(a) con-tribute to atherogenesis through both proatherogenic and prothrombotic mechanisms, mediated by its apolipoprotein(a) moiety, which exhibits structural homology to plasminogen and interferes with fibrinolysis (2, 3). Moreover, the oxidized phospholipids carried by Lp(a) enhance vascular inflammation and endothelial dysfunction, further augmenting atherosclerotic plaque progression (4). Figure 1 shows the cellular pathways of modified LDL uptake and intracellular lipid metabolism.

The clinical relevance of Lp(a) as a cardiovascular risk determinant has been substantiated across large-scale studies. The AIM-HIGH and ODYSSEY OUTCOMES trials demonstrated that elevated Lp(a) concentrations predict future coronary and cardio-vascular events, even among individuals receiving intensive lipid-lowering therapy (5, 6). These findings align with earlier observations showing that Lp(a) levels are genetically determined and only modestly influenced by lifestyle or statin therapy, thereby necessitating novel therapeutic strategies (7). Recent pharmacological interventions, including PCSK9 inhibitors and targeted antisense oligonucleotides such as pelacarsen and muvalaplin, have shown substantial efficacy in reducing Lp(a) concentrations (8, 9).

In parallel, ApoB represents a critical measure of atherogenic particle number, reflecting the total burden of lipoproteins capable of penetrating the arterial wall (10). Elevated ApoB levels are associated with an increased risk of cardiovascular events, irrespective of LDL-C concentrations, particularly among individuals with diabetes mellitus or metabolic syndrome (11). Evidence from controlled trials has demonstrated that ApoB-lowering therapies, including statins, PCSK9 inhibitors, and mipomersen, confer significant reductions in residual cardiovascular risk (12, 13). Furthermore, the ApoB/ApoA-I ratio has been proposed as a superior indicator of cardiometabolic im-balance, integrating pro- and anti-atherogenic lipoprotein activity (14).

The intricate interplay between lipid metabolism, inflammation, and glucose homeostasis further highlights the complexity of cardiometabolic disease. Elevated inter-leukin-6 (IL-6) levels have been shown to mediate dyslipidemic alterations and endothelial injury, while IL-6 inhibition has emerged as a promising anti-inflammatory approach for cardiovascular risk reduction (15). Nutritional and hormonal factors, such as vitamin D supplementation and estrogen therapy, have also demonstrated modulatory effects on Lp(a) and ApoB metabolism (16, 17).

Thus, contemporary evidence positions Lp(a) and ApoB as factors that influence cardiovascular risk landscape, beyond conventional lipid targets. Integrating their evaluation into routine cardiovascular risk assessment may enhance early detection, therapeutic personalization, and outcome prediction. The present study does not aim to redefine the established clinical relevance of Lp(a) and ApoB, both of which are well-recognized markers of atherogenic cardiovascular risk. Instead, our objective was to explore the biochemical and metabolic correlates of Lp(a) and ApoB within a real-world, single-center population, with particular attention to markers of hepatic and renal function.

## 2 Materials and methods

### 2.1 Study design and population

This cross-sectional observational study was conducted on a cohort of adult patients evaluated for cardiometabolic risk profiling at a single tertiary medical center from Mangalia, Romania. The investigation aimed to explore the associations between Lp(a), ApoB, and metabolic as well as cardiovascular parameters in a clinically diverse population. A total of 153 subjects were included after applying eligibility criteria and quality control for complete biochemical and clinical data. The study was conducted in accordance with the Declaration of Helsinki, and approved by the Ethics Committee of Medsim Center (166/23.02.2025).

### 2.2 Inclusion and exclusion criteria

Eligible participants were adults aged 19 to 90 years, of both sexes, who underwent complete fasting biochemical and clinical evaluations. Individuals were included if they had measurable values for Lp(a), ApoB, lipid fractions, glycemic markers, and inflammatory parameters. Exclusion criteria encompassed incomplete datasets, evidence of acute infection, hepatic or renal failure, known malignant disease, or chronic inflammatory or autoimmune disorders that could interfere with lipid metabolism or inflammatory biomarkers. Pregnant women and patients receiving lipid apheresis or investigational lipid-lowering therapies were also excluded.

### 2.3 Clinical and anthropometric assessment

Detailed clinical evaluation was performed for each participant, including anthropometric measurements and cardiovascular risk assessment. Age, sex, smoking status, and medical history (including diabetes mellitus, dyslipidemia, hypertension, and cardiovascular disease) were recorded. Body mass index (BMI) was calculated as weight divided by height squared (kg/m²), and waist circumference (cm) was measured at the midpoint between the lowest rib and the iliac crest. Blood pressure (mmHg) was determined in a seated position after 5 min of rest using a calibrated sphygmomanometer, and the mean of two consecutive readings was used.

### 2.4 Biochemical measurements

Venous blood samples were obtained after a 12 h overnight fast. Hematological parameters were measured using an automated hematology analyzer (Mindray BC-5150) based on electrical impedance, colorimetric methods, and laser flow cytometry, with whole blood samples collected in EDTA tubes. Erythrocyte sedimentation rate (ESR) was determined using the Westergren method from whole blood samples. Fibrinogen concentrations were assessed by coagulometric analysis using the RAYTO RT-2201C analyzer, with serum samples processed under appropriate primary sample conditions. Biochemical parameters, including lipid fractions and renal and hepatic markers, were measured by spectrophotometry using the A25 automated analyzer from serum samples. Hormonal and vitamin assays, including TSH, free thyroxine (FT4), vitamin D, and vitamin B12, were performed using the MAGLUMI X3 chemiluminescence immunoassay system. Lipoprotein(a) concentrations were measured using an automated immunoturbidimetric assay on the same clinical chemistry analyzer used for lipid profile assessment, in accordance with standard laboratory protocols and manufacturer instructions.

All analyses were conducted in the institutional clinical laboratory using commercially available reagents, in accordance with the manufacturers' instructions. Internal quality control procedures were applied routinely, and sample collection, handling, and processing followed standardized laboratory protocols to ensure analytical reliability.

The following parameters were measured:

- Lipid profile: total cholesterol, high-density lipoprotein cholesterol (HDL-C), LDL-C, triglycerides (TG), and Lp(a).
- Apolipoproteins: ApoB quantified by immunoturbidimetric method.
- Inflammatory markers: erythrocyte sedimentation rate (ESR), fibrinogen, and high-sensitivity C-reactive protein (hs-CRP).
- Glycemic parameters: fasting plasma glucose (mg/dL) and glycated hemoglobin (HbA1c, %).
- Renal and hepatic markers: creatinine, urea, uric acid, estimated glomerular filtration rate (eGFR, mL/min/1.73 m²), alanine aminotransferase (ALT), aspartate aminotransferase (AST), and gamma-glutamyl transferase (GGT).
- Electrolytes and micronutrients: serum sodium, potassium, calcium, and magnesium.

Quality control procedures followed international laboratory standards. The coefficients of variation for the lipid parameters were maintained below 5%.

### 2.5 Categorical variables

Categorical variables were coded dichotomously to facilitate statistical analysis:

- Sex (male/female),
- Smoking status (smoker/non-smoker),
- Diabetes mellitus (present/absent),
- Dyslipidemia (present/absent),
- Cardiovascular disease (present/absent),
- Family history of cardiovascular disease (yes/no), and
- Lipid-lowering or antiplatelet treatment (yes/no).

The distribution of categorical data was expressed as absolute frequencies and percentages.

### 2.6 Statistical analysis

All data were processed using IBM SPSS Statistics, version 26.0 (IBM Corp., Armonk, NY, USA) (18). Descriptive statistics were computed for all variables. Continuous variables were tested for normality using the Kolmogorov–Smirnov test and expressed as mean ± standard deviation (SD) for normally distributed data or as median (interquartile range) for non-normal distributions (19). Categorical variables were presented as numbers and percentages.

Pearson's correlation coefficients (20) were calculated to evaluate linear associations between Lp(a), ApoB, and continuous metabolic variables, including BMI, waist circumference, lipid fractions, and inflammatory markers. Pairwise exclusion (21) was ap-plied for missing values. Confidence intervals (95% CI) were calculated for each correlation coefficient. The significance threshold was set at *p* < 0.05 (two-tailed) (22). Lipid-lowering therapy and antiplatelet or anticoagulant treatment were recorded for each participant and analyzed as categorical variables in the statistical evaluation.

Missing value analyses were performed to verify the robustness of the dataset (23). No transformation or outlier exclusion was necessary beyond standard statistical cleaning. The results were graphically represented through scatter plots and correlation matrices to illustrate the direction and strength of relationships between variables.

An exploratory decision tree regression analysis was performed to identify bio-chemical and metabolic variables associated with Lp(a) variability. The model was con-structed using the Exhaustive Chi-square Automatic Interaction Detection (CHAID) algorithm, which iteratively partitions the dataset based on statistically significant as-sociations between predictor variables and the dependent outcome. Lp(a) concentration was specified as the dependent variable. Candidate independent variables included demographic parameters (age, sex), anthropometric indices (BMI, waist circumference), lipid fractions, and markers of hepatic and renal function (including gamma-glutamyl transferase, creatinine, urea, and uric acid).

At each step, node splitting was determined by the highest chi-square statistic with Bonferroni-adjusted *p*-values to control for multiple comparisons. Minimum parent and child node sizes were predefined to avoid overfitting. The decision tree approach was applied as an exploratory, hypothesis-generating tool to visualize nonlinear interactions and to identify potential metabolic factors influencing Lp(a) levels within this cross-sectional cohort.

Normality of continuous variables was assessed using the Kolmogorov–Smirnov test. Variables demonstrating an approximately normal distribution were analyzed using Pearson's correlation coefficient to evaluate linear associations. For variables that deviated from normality, Spearman's rank correlation coefficient was applied. In particular, Lp(a) exhibited a right-skewed distribution and was therefore analyzed using Spearman correlation in non-parametric analyses. The use of Spearman's method was justified by its robustness to non-normal distributions and potential outliers, as it does not assume linearity or normality of the underlying data.

Multivariable linear regression analysis was performed to assess independent as-sociations with Lp(a) levels. Due to its right-skewed distribution, ln-transformed Lp(a) was used as the dependent variable. Independent variables included age, sex, BMI, lipid parameters, and selected hepatic and renal markers, entered simultaneously using the enter method. Model assumptions were evaluated by inspection of residual histograms and normal P–P plots, assessment of homoscedasticity, and variance inflation factors for multicollinearity. Regression coefficients (β), 95% confidence intervals, and two-tailed *p*-values were reported.

## 3 Results

### 3.1 Baseline population characteristics

Tables 1A, B present the descriptive characteristics of the study population (*n* = 153), providing a comprehensive overview of demographic, anthropometric, and biochemical variables. The cohort comprised adults aged between 19 and 90 years, with a mean age of 57.88 ± 12.30 years, reflecting a predominantly middle-aged to elderly population. The mean body mass index was 28.79 ± 4.87 kg/m², indicating a generally overweight profile. Systolic arterial blood pressure values ranged from 100 to 190 mmHg, with an average of 134.02 ± 15.37 mmHg. Detailed baseline clinical, biochemical, and hematological variables included in the analysis are provided in Supplementary Table S1. Also, a comprehensive description of hepatic, renal, metabolic, and endocrine parameters is available in Supplementary Table S2.

**Table 1A:** Population characteristics.

Statistic		Age (years)	BMI (kg/m²)	Blood pressure (mmHg)	Lp(a) (mg/dL)	LDL-C (mg/dL)	HDL-C (mg/dL)	Triglycerides (mg/dL)	Total cholesterol (mg/dL)	ESR	Fibrinogen (mg/dL)	Leukocytes (/µL)	Hemoglobin (g/dL)	Platelets (/µL)	Sodium (mmol/L)
Total	Median	58.00	28.73	130.00	17.00	161.00	61.00	114.00	236.20	10.00	331.20	6,430.00	14.30	2,63,000.00	139.80
	Minimum	19	14.18	100	3	30	42	22.8	99.6	2	200.5	3,860	11.0	86,000	131
	Maximum	90	47.47	190	143	285	82	504.2	364.3	55	572.5	13,300	18.0	4,91,000	146
	Mean	57.88	28.79	134.02	30.14	155.19	61.41	137.17	234.67	13.43	337.07	6,928.76	14.11	2,69,385.62	139.35
	Std. Deviation	12.29	4.86	15.36	31.50	51.38	5.998	89.94	52.50	9.842	81.17	2,000.16	1.34	77,735.476	3.005

**Table 1B:** Population characteristics.

Statistic		AST (U/L)	ALT (U/L)	Creatinine (mg/dL)	Urea (mg/dL)	Uric acid (mg/dL)	Serum Ca (mg/dL)	Mg (mg/dL)	Blood glucose	Glycated Hb	eGFR	CK (UI/mL)	Apo B	WC	TSH (mUI/mL)	GGT
Total	Median	23.09	23.53	0.91	33.00	5.20	9.60	1.94	95.20	5.80	76.64	118.00	117.00	98.00	1.1	98.00
	Minimum	9.83	6.80	0.52	16.4	2.5	8.1	1.6	65	4.8	42.05	34	20	43	0.20	43
	Maximum	343.00	360.20	2.18	66.1	9.4	10.9	3.2	178	9.9	140.14	438	212	135	2.12	135
	Mean	28.76	34.09	0.93	34.63	5.41	9.65	2.00	101.95	6.12	79.2848	136.40	119.87	98.73	1.20	98.33
	Std. Deviation	31.82	38.93	0.21	10.60	1.45	0.65	0.27	22.07	1.15	17.43,529	80.51	36.01	15.87	0.36	15.95

Regarding lipid metabolism, the mean Lp(a) concentration was 30.14 ± 31.50 mg/dL, demonstrating substantial interindividual variability. LDL-C averaged 155.19 ± 51.39 mg/dL, while HDL-C was 61.41 ± 5.99 mg/dL, and triglycerides averaged 137.17 ± 89.95 mg/dL, delineating a mixed dyslipidemic profile. The mean total cholesterol was 234.68 ± 52.50 mg/dL, with ApoB levels averaging 119.87 ± 36.01 mg/dL, consistent with elevated atherogenic particle burden. Inflammatory and hematologic parameters were within reference limits overall, with mean fibrinogen and ESR values of 337.08 ± 81.18 mg/dL and 13.43 ± 9.84 mm/h, respectively. Indicators of hepatic, renal, and metabolic function, including ALT, AST, creatinine, and eGFR, showed preserved organ function across the cohort. The distribution of cardiovascular risk factors and laboratory biomarkers is summarized in Table 1, with extended data available in Supplementary Table S3.

The distribution of Lp(a) concentrations was highly heterogeneous, with values ranging from 3 to 143 mg/dL and a median of 17.0 mg/dL. ApoB concentrations also showed substantial variability (median 117.0 mg/dL; range 20–212 mg/dL). The lipid profile was characterized by elevated total cholesterol (median 236.2 mg/dL) and LDL-C levels (median 161.0 mg/dL), in the presence of relatively preserved HDL-C concentrations (median 61.0 mg/dL). Triglyceride levels exhibited a broad range (22.8–504.2 mg/dL; median 114.0 mg/dL). Inflammatory markers, including fibrinogen (median 331.2 mg/dL) and erythrocyte sedimentation rate (median 10.0 mm/h), were generally within reference limits. Glycemic parameters indicated borderline metabolic control, with median fasting glucose levels of 95.2 mg/dL and a median glycated hemoglobin (HbA1c) of 5.8%. Renal function was largely preserved, as reflected by a median estimated glomerular filtration rate of 76.6 mL/min/1.73 m², although a wide range of values was observed. Full details of patients individual values are included in Supplementary Material.

Table 2 summarizes the categorical variables describing the clinical and therapeutic characteristics of the study population. Of the 153 participants, 43.1% were male and 56.9% were female, indicating a balanced sex distribution with a slight predominance of women. The prevalence of smoking was 24.2%, reflecting a moderate rate of exposure to modifiable behavioral risk factors. Diabetes mellitus was identified in 15.0% of the cohort, while dyslipidemia was the most common comorbidity, present in 79.1% of participants. Cardiovascular diseases, including coronary artery disease, ischemic heart disease, and hypertension, were reported in 75.2% of cases, and 49.7% of individuals had a family history of cardiovascular disease. Regarding pharmacologic management, 66.7% of subjects were on lipid-lowering therapy, predominantly statins, while 73.2% received antiplatelet or anticoagulant treatment.

**Table 2:** Population frequencies.

Variables	Frequency	Percent
Male	66	43.1
Smoking status	37	24.2
Diabetes mellitus	23	15
Dyslipidemia	121	79.1
Cardiovascular diseases	115	75.2
Family history of cardiovascular diseases	76	49.7
Lipid-lowering treatment	102	66.7
Antiplatelet/anticoagulant treatment	112	73.2

The categorical distribution highlights a population with a high cardiovascular risk burden, characterized by a predominance of dyslipidemia and established cardiovascular disease. The relatively high proportion of individuals on lipid-lowering and antithrombotic therapy suggests that most participants were under chronic cardiovascular management, which aligns with the biochemical findings reported in Table 1. The co-existence of dyslipidemia (79.1%) and cardiovascular disease (75.2%) reinforces the relevance of assessing advanced lipid biomarkers such as Lp(a) and ApoB in this clinical context.

The modest prevalence of diabetes mellitus (15%), alongside a nearly 25% rate of smoking, further delineates a multifactorial risk landscape typical of secondary prevention populations. The strong familial aggregation of cardiovascular disease (49.7%) and the wide variability of Lp(a) observed in the biochemical dataset point toward an underlying genetic predisposition influencing lipid metabolism.

**Figure 2:**
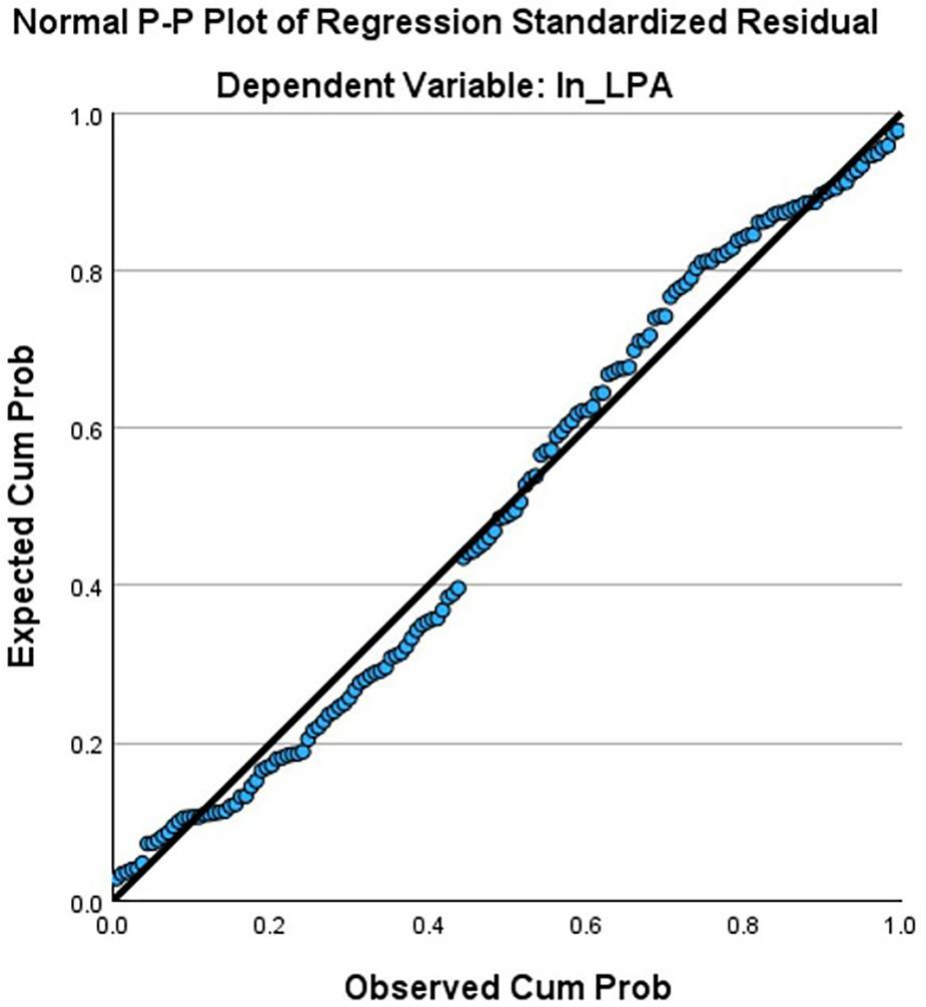
Histogram of standardized residuals from the multivariable regression model with ln-transformed lipoprotein(a) as the dependent variable.

**Figure 3:**
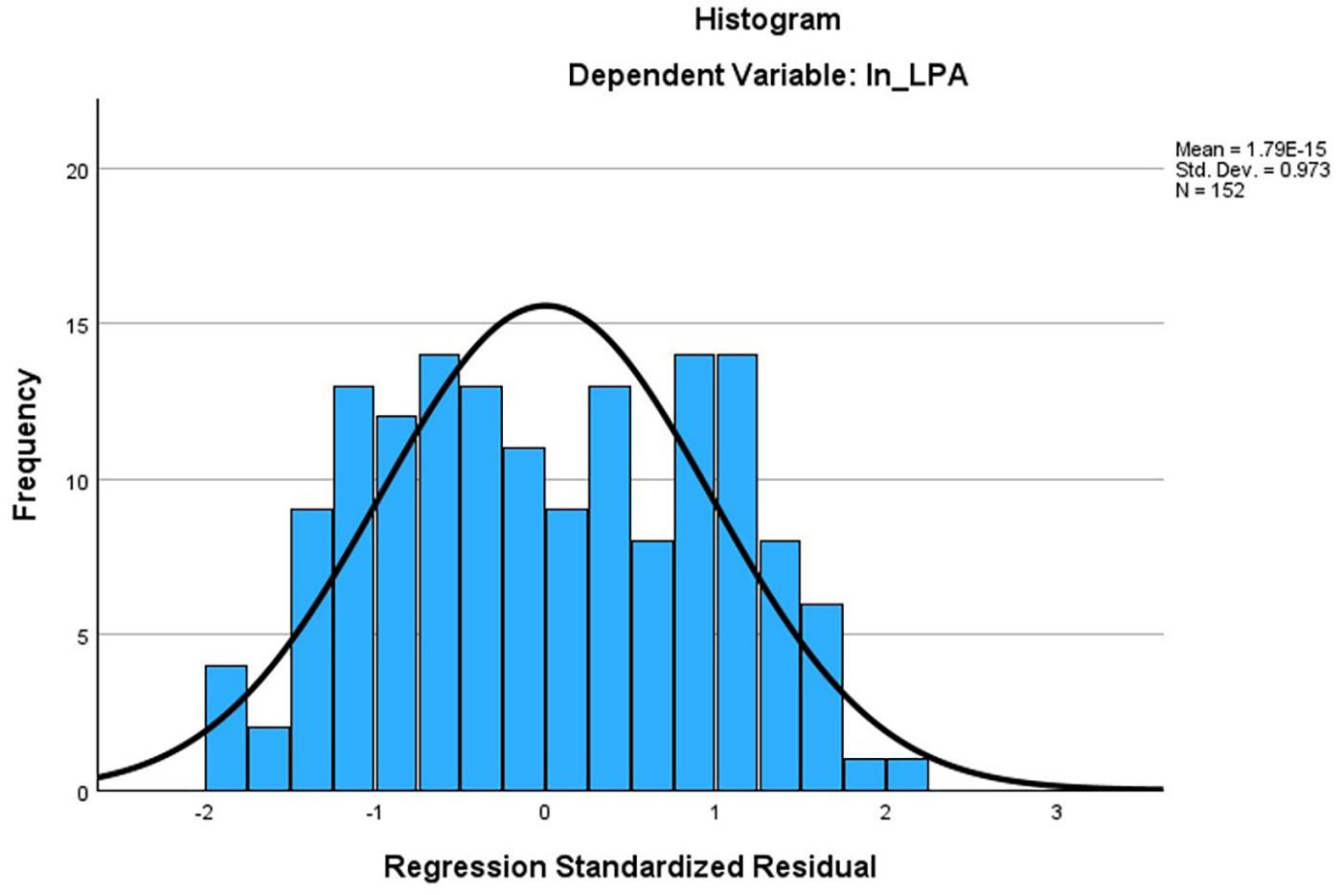
Normal P–P plot of standardized residuals from the multivariable regression model with ln-transformed lipoprotein(a) as the dependent variable. The plot compares the observed cumulative distribution of residuals with the expected normal distribution. The close alignment of points along the diagonal line indicates that the assumption of normality is reasonably satisfied.

Multivariable linear regression analysis was performed with ln-transformed Lp(a) as the dependent variable. Age, sex, BMI, and hepatic and renal function markers were entered simultaneously using the enter method (Figures 2, 3). GGT showed the strongest inverse association with ln-Lp(a), whereas AST and ALT were not independently associated. The distribution of standardized residuals and their normality were evaluated and are presented in Supplementary Figures S1, S2.

The histogram of standardized residuals in Figure 2 demonstrates an approximately normal distribution centered around zero (mean = 1.79 × 10⁻^15^), with a standard deviation of 0.973 across 152 observations. The residuals show no marked skewness or excessive kurtosis, and most values fall within the expected ±2 standard deviation range. Partial regression plots illustrating these associations are provided in Supplementary Figures S3–S7.

The normal P–P plot of standardized residuals from Figure 3 shows a close alignment of the observed cumulative probabilities with the expected normal distribution, with only minor deviations at the distribution tails. The residuals follow the reference diagonal across most of the probability range, indicating that departures from normality are minimal. The adjusted relationships between hepatic and renal parameters and Lp(a) are illustrated in Supplementary Figures S16–S20.

Lp(a) exhibited a right-skewed distribution on initial assessment. Raw Lp(a) values were retained for descriptive statistics and correlation analyses in order to preserve clinical interpretability and avoid distortion of absolute concentration values. No outlier exclusion was performed beyond standard data cleaning procedures.

For multivariable linear regression analyses, in-transformation of Lp(a) was evaluated to improve compliance with model assumptions. The in-transformed variable demonstrated improved residual behavior, with the histogram of standardized residuals showing an approximately normal distribution centered around zero and the normal P–P plot indicating close alignment with the reference diagonal (Figures 2, 3).

**Figure 4:**
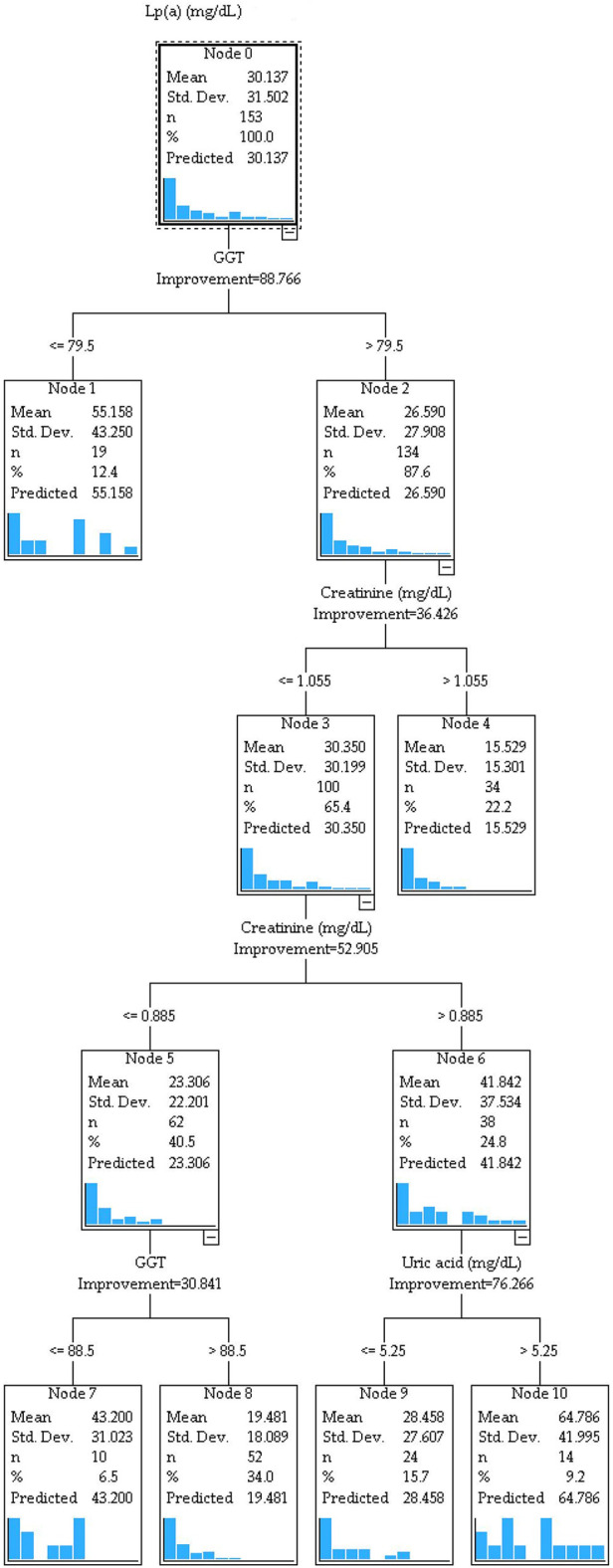
The figure illustrates a classification tree identifying clinical and biochemical variables associated with Lp(a) levels. Each node represents a decision point based on a threshold value of the selected variable, with branches indicating subgroup stratification. Terminal nodes display the mean Lp(a) value within each subgroup. The model highlights the relative importance of variables such as GGT, creatinine, and other metabolic parameters in defining Lp(a) distribution patterns. This analysis is exploratory and intended to identify potential interaction structures rather than establish causal relationships

Figure 4 displays a decision tree regression model constructed to identify the bio-chemical parameters most strongly associated with lipoprotein (a) variability in the studied population. The model was generated using the Exhaustive CHAID algorithm (24), incorporating multiple metabolic and hepatic variables as predictors. The analysis automatically selected GGT, creatinine, and uric acid as the most significant contributors to the variance in Lp(a) concentrations, based on F-statistics and improvement criteria (25). Each node represents a subgroup with a distinct mean Lp(a) value and associated biochemical profile.

The initial split in the model identified GGT as the most influential factor for Lp(a) variability, with a critical threshold of 79.5 U/L yielding the greatest improvement (Improvement = 88.766). Participants with GGT ≤ 79.5 U/L (Node 1) exhibited markedly elevated Lp(a) levels (mean 55.16 ± 43.25 mg/dL) compared with those with higher GGT (>79.5 U/L; Node 2: mean 28.59 ± 27.91 mg/dL).

Within the subgroup with GGT > 79.5 U/L, creatinine emerged as the secondary determinant. Participants with creatinine ≤1.055 mg/dL (Node 3) showed moderate Lp(a) levels (30.35 ± 30.20 mg/dL), whereas those with creatinine >1.055 mg/dL (Node 4) demonstrated considerably lower mean values (15.53 ± 15.30 mg/dL).

Further branching of Node 3 based on creatinine ≤0.885 mg/dL delineated two subgroups (Nodes 5 and 6) with mean Lp(a) values of 23.31 mg/dL and 41.84 mg/dL, respectively.

In the final layer of the tree, GGT and uric acid acted as tertiary discriminators. Among individuals with low creatinine, elevated GGT (>88.5 U/L) was associated with lower Lp(a) concentrations (19.48 mg/dL, Node 8), suggesting hepatic lipoprotein turnover. Conversely, in those with higher creatinine, uric acid >5.25 mg/dL (Node 10) corresponded to the highest Lp(a) levels (64.79 ± 41.99 mg/dL).

A multivariable linear regression model was constructed using ln-transformed Lp(a) as the dependent variable (Table 3). The overall model was not statistically significant (*F* = 1.495, *p* = 0.155), indicating that the selected clinical and biochemical variables did not independently explain variability in Lp(a) levels.

**Table 3:** Multivariable linear regression analysis for ln-transformed Lp(a).

Model	Unstandardized coefficients		Standardized coefficients	t	Sig.	95.0% confidence interval for B	
	B	Std. error	Beta			Lower bound	Upper bound
Age (years)	−.003	.008	−.037	−.409	.683	−.019	.013
Male	−.323	.204	−.144	−1.584	.115	−.726	.080
BMI (kg/m²)	.056	.029	.245	1.954	.053	−.001	.112
AST (U/L)	.003	.008	.092	.427	.670	−.012	.018
ALT (U/L)	−.005	.006	−.179	−.802	.424	−.018	.008
GGT	−.017	.009	−.251	−1.841	.068	−.036	.001
Creatinine (mg/dL)	.091	.470	.017	.194	.847	−.838	1.021
Uric acid (mg/dL)	−.042	.063	−.055	−.673	.502	−.166	.082

None of the included variables reached statistical significance. However, BMI (*B* = 0.056, 95% CI: −0.001 to 0.112, *p* = 0.053) and GGT (*B* = −0.017, 95% CI: −0.036 to 0.001, *p* = 0.068) demonstrated borderline associations, with GGT showing a trend toward an inverse relationship with ln-Lp(a).

Table 4 summarizes the correlation coefficients between Lp(a), ApoB, and a series of anthropometric, lipid, and cardiovascular variables in the study cohort. A modest but significant inverse correlation is observed between Lp(a) with waist circumference (*r* = –0.172; 95% CI: −0.322 to −0.013), suggesting that abdominal adiposity may be associated with slightly lower Lp(a) levels. No meaningful associations were detected between Lp(a) and LDL-C (*r* = 0.075) or triglycerides (*r* = –0.144).

**Table 4:** Correlation coefficients between lipoprotein(a), apolipoprotein B, and clinical–metabolic parameters.

Biomarkers	Variables	Correlation	Count	Lower C.I.	Upper C.I.
Lp(a)	BMI	−.041	153	−.198	.119
	Dyslipidemia	.044	153	−.115	.201
	Cardiovascular diseases	−.125	153	−.278	.035
	LDL	.075	153	−.085	.231
	HDL	−.034	153	−.191	.126
	Triglycerides	−.144	153	−.296	.015
	Total cholesterol	.040	153	−.119	.198
	Waist circumference	−.172	153	−.322	−.013
ApoB	BMI	.093	153	−.066	.248
	Dyslipidemia	.150	153	−.009	.302
	Cardiovascular diseases	−.192	153	−.340	−.035
	LDL	.264	153	.110	.406
	HDL	−.112	153	−.266	.047
	Triglycerides	.460	153	.326	.577
	Total cholesterol	.492	153	.361	.603
	Waist circumference	.051	153	−.109	.208

Adjusted for age, sex, BMI, lipid profile parameters (LDL-C, HDL-C, triglycerides), and hepatic and renal function markers (AST, ALT, GGT, creatinine, uric acid).

In contrast, ApoB exhibited strong positive correlations with total cholesterol (*r* = 0.492; 95% CI: 0.361–0.603) and triglycerides (*r* = 0.460; 95% CI: 0.326–0.577), as well as a moderate correlation with LDL-C (*r* = 0.264; 95% CI: 0.110–0.406).

Table 5 summarizes the correlation analyses between LDL-C, Lp(a), ApoB, and the use of lipid-lowering and antiplatelet/anticoagulant therapies. Correlations were calculated using pairwise exclusion of missing values, with 95% confidence intervals reported for each association. LDL-C showed a weak inverse correlation with lipid-lowering treatment (*r* = −0.147; 95% CI: −0.299 to 0.012), while no significant association was observed with antiplatelet or anticoagulant therapy. Lp(a) levels were not correlated with lipid-lowering (*r* = −0.055; 95% CI: −0.212 to 0.104) or antithrombotic treatment (*r* = −0.032; 95% CI: −0.190 to 0.127). Similarly, ApoB showed no significant association with either lipid-lowering therapy (*r* = 0.071; 95% CI: −0.089 to 0.227) or antiplatelet/anticoagulant use. Model diagnostics, including relationships between predicted values, residuals, and influence statistics, are shown in Supplementary Figures S10–S15.

**Table 5:** Correlations between lipid-related biomarkers and pharmacological treatment status.

Correlations					
		Statistic			
Variable	Variable2	Correlation	Count	Lower C.I.	Upper C.I.
LDL	Lipid lowering treatment	−.147	153	−.299	.012
	Antiplatelet anticoagulant treatment	−.120	153	−.274	.039
LPA	Lipid lowering treatment	−.055	153	−.212	.104
	Antiplatelet anticoagulant treatment	−.032	153	−.190	.127
LPB	Lipid lowering treatment	.071	153	−.089	.227
	Antiplatelet anticoagulant treatment	−.009	153	−.168	.150

Missing value handling: PAIRWISE, EXCLUDE. C.I. Level: 95.0/Adjusted for age, sex, BMI, lipid profile parameters (LDL-C, HDL-C, triglycerides), and hepatic and renal function markers (AST, ALT, GGT, creatinine, uric acid).

SupplementaryTables S4, S5 from the Supplementary Material present Pearson and Spearman correlation analyses exploring the relationships between lipoprotein(a) [Lp(a)], anthropometric parameters, lipid fractions, and cardiovascular disease status. Both para-metric and non-parametric approaches were applied to account for potential deviations from normality, particularly for Lp(a). Across both correlation methods, Lp(a) showed no significant association with body mass index, LDL-C, HDL-C, triglycerides, or total cholesterol, supporting its relative independence from conventional lipid parameters. In contrast, expected correlations were observed among classical lipid variables, including strong positive associations between LDL-C and total cholesterol, and inverse relation-ships between HDL-C and triglycerides, confirming the internal consistency and bio-logical plausibility of the dataset.

Supplementary regression models were constructed to further explore potential Lp(a) variability. In the first multivariable model, demographic and metabolic variables (sex, BMI, diabetes mellitus, LDL-C, and triglycerides) explained a limited proportion of Lp(a) variance, with no independent predictors reaching statistical significance. A second model focusing on hepatic and renal markers similarly demonstrated modest explanatory power; however, gamma-glutamyl transferase emerged as a significant inverse predictor of Lp(a) levels. These supplementary regression analyses were conducted as exploratory evaluations and should be interpreted cautiously, serving to complement the primary correlation and decision tree findings rather than to infer causality (Supplementary Material).

## 4 Discussions

The present study highlights a complex relationship between Lp(a), ApoB, and markers of hepatic and renal function, emphasizing their interconnected roles in cardiometabolic risk. The observed heterogeneity of Lp(a) concentrations (range: 3–143 mg/dL) supports the notion that this lipoprotein is primarily genetically determined, as previously established in population-based studies. Similar to our results, Gravholt et al. (17) demonstrated that hormonal changes can influence lipoprotein metabolism, suggesting that genetic and endocrine factors jointly regulate circulating Lp(a) and ApoB levels.

One of the most notable findings of this study was the inverse association between GGT levels and Lp(a), with patients exhibiting lower GGT values demonstrating higher Lp(a) concentrations. This direction of association is not intuitively expected and should be interpreted with caution. Several potential explanations may be considered. First, GGT is widely recognized as a marker of hepatic metabolic activity and oxidative stress rather than a direct regulator of lipoprotein synthesis. It is possible that lower GGT levels reflect a distinct metabolic profile characterized by reduced hepatic turnover or altered redox balance, which may indirectly influence lipoprotein metabolism. Second, GGT is closely associated with components of the metabolic syndrome, including insulin resistance and triglyceride-rich lipoproteins. In this context, higher GGT levels may be linked to metabolic states that preferentially affect other lipid fractions (e.g., triglycerides or ApoB-containing particles), without directly increasing Lp(a), which is largely genetically determined. The inverse association between GGT and Lp(a) remained consistent across adjusted analyses and is visually supported by the partial regression plot (Supplementary Figure S18), which does not suggest distortion by outliers.

Although alcohol consumption is a known determinant of GGT levels and has been reported to reduce Lp(a) concentrations in some studies, this variable was not systematically assessed in our cohort. Therefore, its potential contribution cannot be quantified and should be considered a possible but unverified source of residual confounding.

Importantly, this association did not persist as an independent predictor in multivariable analysis, further supporting the interpretation that it may reflect residual confounding rather than a direct biological effect. Therefore, the inverse relationship between GGT and Lp(a) observed in this cohort should be regarded as hypothesis-generating and warrants confirmation in larger studies with detailed metabolic and lifestyle characterization.

Consistent with our findings showing an association between elevated ApoB and higher total cholesterol and triglyceride levels, Rosenblit et al. (12) emphasized ApoB as a superior indicator of atherogenic particle burden compared with LDL-C alone, particularly in high-risk individuals. Similarly, Leiter et al. (26) confirmed that therapies targeting PCSK9, such as inclisiran, substantially reduce LDL-C and ApoB levels regardless of diabetes status.

The observed inverse association between Lp(a) and waist circumference is somewhat unexpected, given the established relationship between central obesity and adverse lipid profiles. However, Lp(a) differs from other lipoproteins in that its levels are largely genetically determined and relatively independent of metabolic factors such as adiposity and insulin resistance. It is therefore possible that this inverse relationship reflects residual confounding or the influence of unmeasured variables rather than a direct biological effect. Additionally, metabolic disturbances associated with increased waist circumference may predominantly affect triglyceride-rich lipoproteins and ApoB-containing particles, with limited direct impact on Lp(a) concentrations.

In our study, hepatic enzymes, especially GGT, emerged as factors of Lp(a) variability. This is concordant with evidence linking hepatic metabolic activity to lipoprotein synthesis and turnover. Lorenzatti et al. (27) reported similar associations in patients with type 2 diabetes and dyslipidemia treated with evolocumab, demonstrating that hepatic lipid metabolism remains central to Lp(a) regulation even under intensive lipid-lowering therapy. Furthermore, Chiva-Blanch et al. (28) highlighted that moderate intake of polyphenol-rich red wine improved lipid and glucose metabolism.

A recent review emphasized that ApoB reflects the total number of circulating atherogenic lipoprotein particles, whereas Lp(a) represents an additional genetically determined and independently proatherogenic lipoprotein fraction associated with inflammation, thrombosis, and accelerated atherosclerosis. The authors highlighted that combined assessment of ApoB and Lp(a) may improve residual cardiovascular risk identification beyond conventional lipid parameters, particularly in patients with dyslipidemia and established cardiovascular disease receiving lipid-lowering therapy (27). These observations are consistent with our findings, in which ApoB showed strong correlations with total cholesterol and triglycerides, while Lp(a) demonstrated substantial interindividual variability and relative independence from conventional lipid fractions. In a multiple logistic regression analysis for median Lp(a), Hiraishi et al. showed that there was an inverse association between eGFR and Lp(a) level (odds ratio, 0.965; 95% confidence interval, 0.935–0.997; *P* = 0.030) in non-statin users as well as in all participants, but not in statin users (29).

According to Kronenberg et al. Lp(a) levels begin to increase in the earliest stages of renal impairment before GFR starts to decrease, thus lipoprotein(a) plasma levels are markedly influenced by the presence of chronic kidney disease and the GFR (30).

In our exploratory decision-tree analysis, higher creatinine values were associated with lower Lp(a) concentrations within a specific branch of the model. This inverse direction does not support a simple interpretation based on reduced renal clearance of lipoproteins and should therefore be interpreted cautiously. Although renal dysfunction has been associated with altered Lp(a) levels in other clinical settings, our findings do not demonstrate an independent positive relationship between creatinine and Lp(a). In the present cohort, creatinine was not significantly associated with ln-transformed Lp(a) in multivariable regression. The relationship between creatinine and Lp(a), illustrated in Supplementary Figure S19, shows a dispersed pattern without a clear linear trend, supporting the lack of statistical significance observed in the regression model. Comparable patterns were described by Hernández-Mijares et al. (31), who observed metabolic improvements following lipid-modifying interventions in patients with altered carbohydrate metabolism, emphasizing that renal function indirectly shapes lipoprotein profiles. Elevated uric acid, which in our study corresponded with higher Lp(a) levels, denote increased oxidative stress, consistent with the findings of Yadav et al. (32), who demonstrated that inflammatory mediators and oxidative stress impair HDL functionality and may also influence Lp(a) dynamics.

The mean ApoB concentration in our cohort (119.87 ± 36.01 mg/dL) reflects an in-creased number of atherogenic particles, aligning with Criqui et al. (33), who stressed the prognostic importance of identifying individuals at risk for peripheral arterial disease based on subclinical lipid alterations. Additionally, our observation of elevated total cholesterol and triglycerides mirrors the lipid pattern described by Gravholt et al. (17) and by Lorenzatti et al. (27) in dyslipidemic and diabetic populations.

The present findings support the concept that hepatic and renal metabolic parameters are associated with interindividual variability in Lp(a) levels, while ApoB remains a robust indicator of overall atherogenic lipoprotein burden. Although these relationships are well established, their concurrent evaluation may provide complementary information when interpreted alongside traditional lipid indices. In line with previous re-ports by Rosenblit (12) and Leiter et al. (26), the assessment of Lp(a) and ApoB may contribute to a more refined characterization of residual cardiovascular risk, particularly in patients receiving lipid-lowering therapy.

Also, the lack of a significant association between creatinine and Lp(a) levels contrasts with previously proposed mechanisms linking renal function to lipoprotein metabolism. While renal impairment has been associated with altered Lp(a) concentrations in certain populations, our results did not support an independent relationship after adjustment (34).

Blood pressure and glucose-related parameters were not included in the multivariable model to avoid overfitting and multicollinearity, given the sample size and the inclusion of closely related metabolic variables such as BMI and lipid parameters (35–37).

This study has several limitations that should be acknowledged. First, the relatively modest sample size may have limited the statistical power to detect weaker associations. Second, the cross-sectional design precludes causal inference regarding the observed relationships between Lp(a), ApoB, and metabolic parameters. Third, the study was conducted in a single tertiary medical center, which may limit the generalizability of the findings to broader populations. In addition, Lp(a) exhibits substantial interindividual variability, largely driven by genetic factors as well as by age- and sex-related influence. It should also be acknowledged that Lp(a) exhibits substantial interindividual variability, largely driven by genetic factors as well as by age- and sex-related factors. Consequently, robust population-level inference regarding Lp(a) regulation typically requires large, genetically characterized cohorts. Larger, multicenter studies incorporating genetic profiling are war-ranted to validate and extend these observations.

## 5 Conclusions

In this single-center cross-sectional study, Lp(a) levels were not independently associated with the evaluated clinical and biochemical parameters after multivariable adjustment. Although exploratory analyses suggested potential trends involving GGT and BMI, these did not reach statistical significance and should be interpreted with caution.

Among the 153 participants analyzed, the mean Lp(a) level was 30.14 ± 31.50 mg/dL, while ApoB averaged 119.87 ± 36.01 mg/dL. The mean LDL-C concentration of 155.19 ± 51.39 mg/dL and total cholesterol of 234.68 ± 52.50 mg/dL reflected persistent lipid elevation despite lipid-lowering therapy in two-thirds of patients (66.7%).

Correlation analyses revealed that ApoB was strongly associated with total cholesterol (*r* = 0.49, *p* < 0.001) and triglycerides (*r* = 0.46, *p* < 0.001), featuring its role as an integrated marker of atherogenic particle load. Lp(a), although demonstrating weaker correlations with lipid fractions, exhibited significant associations with waist circumference (*r* = –0.17, *p* = 0.04) and showed biochemical changes by hepatic and renal function markers.

These findings show the limited predictive value of routine laboratory markers for Lp(a) variability and support the concept that Lp(a) is largely genetically determined. Further studies incorporating genetic profiling and detailed metabolic characterization are needed to better understand the determinants of Lp(a) levels in patients with cardiovascular disease.

## Data Availability

The original contributions presented in the study are included in the article/Supplementary Material, further inquiries can be directed to the corresponding author/s.

## References

[1] O'Donoghue ML Fazio S Giugliano RP Stroes ESG Kanevsky E Gouni-Berthold I. Lipoprotein(a), PCSK9 inhibition, and cardiovascular risk. Circulation. (2019) 139(12):1483–92. 10.1161/CIRCULATIONAHA.118.037184 30586750

[2] Tsimikas S Karwatowska-Prokopczuk E Gouni-Berthold I Tardif JC Baum SJ Steinhagen-Thiessen E. Lipoprotein(a) reduction in persons with cardiovascular disease. N Engl J Med. (2020) 382(3):244–55. 10.1056/NEJMoa1905239 31893580

[3] Fraley AE Schwartz GG Olsson AG Kinlay S Szarek M Rifai N. Relationship of oxidized phospholipids and biomarkers of oxidized low-density lipoprotein with cardiovascular risk factors, inflammatory biomarkers, and effect of statin therapy in patients with acute coronary syndromes: results from the MIRACL (myocardial ischemia reduction with aggressive cholesterol lowering) trial. J Am Coll Cardiol. (2009) 53(23):2186–96. 10.1016/j.jacc.2009.02.041 19497447

[4] Nestel PJ Barnes EH Tonkin AM Simes J Fournier M White HD. Plasma lipoprotein(a) concentration predicts future coronary and cardiovascular events in patients with stable coronary heart disease. Arterioscler Thromb Vasc Biol. (2013) 33(12):2902–8. 10.1161/ATVBAHA.113.302479 24092750

[5] Albers JJ Slee A O'Brien KD Robinson JG Kashyap ML Kwiterovich PO Jr Relationship of apolipoproteins A-1 and B, and lipoprotein(a) to cardiovascular outcomes: the AIM-HIGH trial (atherothrombosis intervention in metabolic syndrome with low HDL/high triglyceride and impact on global health outcomes). J Am Coll Cardiol. (2013) 62(17):1575–9. 10.1016/j.jacc.2013.06.051 23973688 PMC3800510

[6] ClinicalTrials.gov. ODYSSEY Outcomes: Evaluation of cardiovascular outcomes after an acute coronary syndrome during treatment with alirocumab (NCT01663402). North America, Europe, Latin America, and the Asia-Pacific region: U.S. National Library of Medicine (2019). Available online at: https://clinicaltrials.gov/study/NCT01663402 (Accessed February 11, 2026).

[7] Stone NJ Smith SC Jr Orringer CE Rigotti NA Navar AM Khan SS. Managing atherosclerotic cardiovascular risk in young adults: JACC state-of-the-art review. J Am Coll Cardiol. (2022) 79(8):819–36. 10.1016/j.jacc.2021.12.016 35210038

[8] Nissen SE Wolski K Watts GF Koren MJ Fok H Nicholls SJ. Single ascending and multiple-dose trial of zerlasiran, a short interfering RNA targeting lipoprotein(a): a randomized clinical trial. JAMA. (2024) 331(18):1534–43. 10.1001/jama.2024.4504 38587822 PMC11002768

[9] Colhoun HM Leiter LA Müller-Wieland D Cariou B Ray KK Tinahones FJ. Effect of alirocumab on individuals with type 2 diabetes, high triglycerides, and low high-density lipoprotein cholesterol. Cardiovasc Diabetol. (2020) 19(1):14. 10.1186/s12933-020-0991-1 32035487 PMC7007683

[10] Thomas GS Cromwell WC Ali S Chin W Flaim JD Davidson M. Mipomersen, an apolipoprotein B synthesis inhibitor, reduces atherogenic lipoproteins in patients with severe hypercholesterolemia at high cardiovascular risk: a randomized, double-blind, placebo-controlled trial. J Am Coll Cardiol. (2013) 62(23):2178–84. 10.1016/j.jacc.2013.07.081 24013058

[11] Creager MA Barnes GD Giri J Mukherjee D Jones WS Burnett AE. 2026 AHA/ACC/ACCP/ACEP/CHEST/SCAI/SHM/SIR/SVM/SVN guideline for the evaluation and management of acute pulmonary embolism in adults: a report of the American college of cardiology/American heart association joint committee on clinical practice guidelines. Circulation. (2026) 153(12):e977–e1051. 10.1161/CIR.0000000000001415 41712677

[12] Rosenblit PD. Lowering targeted atherogenic lipoprotein cholesterol goals for patients at “Extreme” ASCVD risk. Curr Diab Rep. (2019) 19(12):146. 10.1007/s11892-019-1246-y 31754844

[13] Ooi EM Watts GF Chan DC Pang J Tenneti VS Hamilton SJ. Effects of extended-release niacin on the postprandial metabolism of Lp(a) and ApoB-100-containing lipoproteins in statin-treated men with type 2 diabetes mellitus. Arterioscler Thromb Vasc Biol. (2015) 35(12):2686–93. 10.1161/ATVBAHA.115.306136 26515419

[14] Kadoglou NP Fotiadis G Athanasiadou Z Vitta I Lampropoulos S Vrabas IS. The effects of resistance training on ApoB/ApoA-I ratio, Lp(a) and inflammatory markers in patients with type 2 diabetes. Endocrine. (2012) 42(3):561–9. 10.1007/s12020-012-9650-y 22407494

[15] Chertow GM Chang AM Felker GM Heise M Velkoska E Fellström B. IL-6 inhibition with clazakizumab in patients receiving maintenance dialysis: a randomized phase 2b trial. Nat Med. (2024) 30:2328–36. 10.1038/s41591-024-03043-1 38796655 PMC11333272

[16] Schwetz V Scharnagl H Trummer C Stojakovic T Pandis M Grübler MR. Vitamin D supplementation and lipoprotein metabolism: a randomized controlled trial. J Clin Lipidol. (2018) 12(3):588–596.e4. 10.1016/j.jacl.2018.03.079 29653812

[17] Højbjerg Gravholt C Christian Klausen I Weeke J Sandahl Christiansen J. Lp(a) and lipids in adult Turner’s syndrome: impact of treatment with 17beta-estradiol and norethisterone. Atherosclerosis. (2000) 150(1):201–8. 10.1016/S0021-9150(99)00369-X 10781652

[18] Sen S Yildirim I. A tutorial on how to conduct meta-analysis with IBM SPSS statistics. Psych. (2022) 4(4):640–67. 10.3390/psych4040049

[19] Cardoso DO Galeno TD. Online evaluation of the kolmogorov–smirnov test on arbitrarily large samples. J Comput Sci. (2023) 67:101959. 10.1016/j.jocs.2023.101959

[20] Dufera AG Liu T Xu J. Regression models of Pearson correlation coefficient. Stat Theory Relat Fields. (2023) 7(2):97–106. 10.1080/24754269.2023.2164970

[21] Kim KH Kim KJ. Missing-data handling methods for lifelogs-based wellness Index estimation: comparative analysis with panel data. JMIR Med Inform. (2020) 8(12):e20597. 10.2196/20597 33331831 PMC7775200

[22] Røislien J. Saving lives with statistics. Scand J Trauma Resusc Emerg Med. (2024) 32(1):79. 10.1186/s13049-024-01256-4 39223573 PMC11370087

[23] Somer E Gische C Miočević M. Methods for modeling autocorrelation and handling missing data in mediation analysis in single case experimental designs (SCEDs). Eval Health Prof. (2022) 45(1):36–53. 10.1177/01632787211071136 35225017 PMC8980456

[24] Miller B Fridline M Liu PY Marino D. Use of CHAID decision trees to formulate pathways for the early detection of metabolic syndrome in young adults. Comput Math Methods Med. (2014) 2014:242717. 10.1155/2014/242717 24817904 PMC4003739

[25] Sureiman O Mangera CM. F-Test of overall significance in regression analysis simplified. J Pract Cardiovasc Sci. (2020) 6(2):116–22. 10.4103/jpcs.jpcs_18_20

[26] Leiter LA Teoh H Kallend D Wright RS Landmesser U Wijngaard PLJ. Inclisiran lowers LDL-C and PCSK9 irrespective of diabetes status: the ORION-1 randomized clinical trial. Diabetes Care. (2019) 42(1):173–6. 10.2337/dc18-1491 30487231

[27] Lorenzatti AJ Eliaschewitz FG Chen Y Lu J Baass A Monsalvo ML. Randomised study of evolocumab in patients with type 2 diabetes and dyslipidaemia on background statin: primary results of the BERSON clinical trial. Diabetes Obes Metab. (2019) 21(6):1455–63. 10.1111/dom.13680 30821053 PMC6594020

[28] Chiva-Blanch G Urpi-Sarda M Ros E Valderas-Martinez P Casas R Arranz S. Effects of red wine polyphenols and alcohol on glucose metabolism and the lipid profile: a randomized clinical trial. Clin Nutr. (2013) 32(2):200–6. 10.1016/j.clnu.2012.08.022 22999066

[29] Hiraishi C Matsui S Kojima T Sato R Ando K Fujimoto K. Association of renal function and statin therapy with lipoprotein(a) in patients with type 2 diabetes. J Atheroscler Thromb. (2023) 31(1):81–9. 10.5551/jat.64261 37558461 PMC10776332

[30] Kronenberg F Utermann G. Lipoprotein(a): resurrected by genetics (review). J Intern Med. (2012) 273:6–30. 10.1111/j.1365-2796.2012.02592.x 22998429

[31] Hernandez-Mijares A Lluch I Vizcarra E Martínez-Triguero ML Ascaso JF Carmena R. Ciprofibrate effects on carbohydrate and lipid metabolism in type 2 diabetes mellitus subjects. Nutr Metab Cardiovasc Dis. (2000) 10(1):1–6. 10812581

[32] Yadav R Liu Y Kwok S Hama S France M Eatough R. Effect of extended-release niacin on high-density lipoprotein functionality, lipoprotein metabolism, and mediators of vascular inflammation in statin-treated patients. J Am Heart Assoc. (2015) 4(9):e001508. 10.1161/JAHA.114.001508 26374297 PMC4599486

[33] Criqui MH Denenberg JO Langer RD Fronek A. The epidemiology of peripheral arterial disease: importance of identifying the population at risk. Vasc Med. (1997) 2(3):221–6. 10.1177/1358863X9700200310 9546971

[34] Scientific image and illustration software | BioRender. (n.d.) Available online at: https://www.biorender.com/ (Accessed March 31, 2026).

[35] Sheldon MR Fillyaw MJ Thompson WD. The use and interpretation of the friedman test in the analysis of ordinal-scale data in repeated measures designs. Physiother Res Int. (1996) 1(4):221–8. 10.1002/pri.66 9238739

[36] Kohler J Hur KH Mueller JD. Statistical analysis of the autocorrelation function in fluorescence correlation spectroscopy. Biophys J. (2024) 123(6):667–80. 10.1016/j.bpj.2024.01.011 38219016 PMC10995414

[37] Alwateer M Atlam ES El-Raouf MMA Ghoneim OA Gad I. Missing data imputation: a comprehensive review. J Comput Commun. (2024) 12(11):53–75. 10.4236/jcc.2024.1211004

