# Supplementary material for: Clinical correlates of lipoprotein (a) and apolipoprotein B levels in patients with dyslipidemia and cardiovascular disease
Source: Front Cardiovasc Med. 2026 Jul 9;13:1821490. doi: 10.3389/fcvm.2026.1821490 (PMC13391904; doi:10.3389/fcvm.2026.1821490)

SUPPLEMENTARY MATERIAL

Table S1. Baseline clinical, biochemical, and hematological characteristics of the study population

		Age (years)	BMI (kg/m²)	BP (mmHg)	Lp(a) (mg/dL)	LDL-C (mg/dL)	HDL-C (mg/dL)	Triglycerides (mg/dL)	TC (mg/dL)	ESR	Fibrinogen (mg/dL)	Leukocytes (/µL)	Hemoglobin (g/dL)	Platelets (/µL)	Sodium (mmol/L)
Total	Median	58.00	28.7300	130.00	17.00	161.00	61.00	114.000	236.200	10.00	331.200	6430.00	14.300	263000.00	139.80
	Minimum	19	14.18	100	3	30	42	22.8	99.6	2	200.5	3860	11.0	86000	131
	Maximum	90	47.47	190	143	285	82	504.2	364.3	55	572.5	13300	18.0	491000	146
	Mean	57.88	28.7927	134.02	30.14	155.19	61.41	137.171	234.676	13.43	337.078	6928.76	14.110	269385.62	139.35
	SD	12.296	4.86619	15.369	31.502	51.389	5.998	89.9492	52.5017	9.842	81.1751	2000.160	1.3451	77735.476	3.005

Table S2. Descriptive statistics of hepatic, renal, metabolic, and endocrine laboratory parameters in the study population

		AST	ALT	Creatinine	Urea	Uric	Serum Ca	Mg	Glucose	Glycated Hb	eGFR	CK	Apo B	WC	TSH	GGT
Total	Median	23.0900	23.5350	0.9100	33.000	5.200	9.600	1.940	95.20	5.800	76.6400	118.00	117.00	98.00	1.1700	98.00
	Minimum	9.83	6.80	0.52	16.4	2.5	8.1	1.6	65	4.8	42.05	34	20	43	0.20	43
	Maximum	343.00	360.20	2.18	66.1	9.4	10.9	3.2	178	9.9	140.14	438	212	135	2.12	135
	Mean	28.7655	34.0971	0.9360	34.631	5.412	9.650	2.008	101.95	6.123	79.2848	136.40	119.87	98.73	1.2048	98.33
	SD	31.82244	38.93993	0.21038	10.6042	1.4578	0.6587	0.2710	22.072	1.1517	17.43529	80.514	36.013	15.872	0.36328	15.955

a. Limited to first 300 cases.

Table S3. Distribution of cardiovascular risk factors and laboratory biomarkers in the study population

	Age (years)	BMI (kg/m²)	BP (mmHg)	Lp(a) (mg/d)	LDL-C (mg/dL)	HDL-C (mg/dL)	Triglycerides (mg/dL)	TC (mg/dL)	ESR	Fibrinogen (mg/dL)	Leukocytes (/µL)	Hemoglobin (g/dL)	Platelets (/µL)	Sodium (mmol/L)
1	70	28.35	130	77	215	62	131.8	303.2	30	331.2	5910	16.5	254000	138
2	68	32.28	150	7	150	63	103.3	234.1	30	301.8	8200	13.8	215000	141
3	68	29.36	130	18	141	62	95.2	222.2	25	372.3	5380	14.4	491000	138
4	60	37.80	130	83	211	61	118.0	295.8	30	437.0	9900	14.0	338000	139
5	41	24.82	110	80	141	72	186.2	296.2	5	215.9	6290	13.4	284000	135
6	25	18.44	110	103	211	61	83.9	222.2	2	276.8	5990	13.3	223000	141
7	45	18.03	110	107	161	57	112.3	240.0	30	247.7	9900	13.2	393000	138
8	22	21.50	110	24	161	59	144.8	186.1	5	236.4	5910	12.8	272000	139
9	37	33.00	110	86	140	66	73.8	221.2	5	356.0	5820	15.2	264000	139
10	50	23.53	145	3	161	72	83.9	222.2	25	527.6	6090	13.6	327000	142
11	36	33.00	145	80	150	61	112.3	222.2	30	476.2	4460	15.0	284000	139
12	49	21.33	120	104	206	63	56.5	284.0	10	256.0	5760	15.6	286000	139
13	26	21.80	115	80	150	57	23.2	185.0	2	253.4	6430	14.5	159000	142
14	52	26.70	145	121	203	61	80.6	280.3	5	265.0	5330	16.9	206000	139
15	55	23.53	120	80	171	61	49.3	291.0	10	324.0	5570	14.2	297000	131
16	24	25.82	125	24	93	72	83.9	181.4	2	218.3	6090	12.7	264000	133
17	54	32.24	120	3	165	61	112.3	248.2	5	276.8	4460	15.0	289000	140
18	64	33.69	135	80	189	57	144.8	290.9	2	247.7	6090	14.9	327000	141
19	60	24.91	125	39	191	59	91.1	268.3	5	236.4	4460	13.3	159000	138

20	66	23.12	135	4	168	66	54.6	244.6	10	356.0	6090	16.9	206000	139
21	57	26.03	115	75	118	65	111.8	205.0	55	527.6	4460	14.2	321000	131
22	58	31.71	140	87	205	45	344.0	300.0	30	476.2	7520	14.6	227000	133
23	54	23.53	145	78	112	62	158.1	205.6	10	247.7	9860	12.8	425000	140
24	54	47.47	145	31	247	58	131.7	331.4	25	452.1	7200	13.3	145000	136
25	78	32.99	160	34	59	63	137.1	149.8	10	255.9	4890	11.7	425000	142
26	76	34.09	120	33	30	64	199.1	134.3	20	382.1	10520	13.2	296000	144
27	66	30.82	130	46	53	61	180.2	149.5	5	265.0	10540	16.5	254000	142
28	36	28.09	160	3	210	61	85.5	288.0	4	315.6	5150	13.8	215000	136
29	54	26.18	145	55	179	58	67.1	258.6	20	316.3	7300	14.4	491000	136
30	58	28.41	130	5	81	62	95.2	161.6	10	231.8	8360	14.0	434000	138
31	61	28.12	145	54	143	63	183.3	243.1	20	346.6	7900	13.9	220000	140
32	66	27.34	100	12	214	72	132.4	312.7	17	433.4	5390	15.4	299000	142
33	57	21.07	120	42	213	61	164.8	307.1	10	395.2	10520	15.4	349000	141
34	47	24.46	140	143	197	62	108.7	280.3	15	254.2	5160	15.4	245000	139
35	64	29.76	120	52	94	70	58.8	176.2	2	253.4	5740	14.4	322000	142
36	48	32.60	145	5	213	52	428.8	211.4	8	545.6	9160	14.7	254000	142
37	53	29.59	120	42	263	58	216.2	364.2	50	371.6	6040	15.1	410000	138
38	55	32.78	140	7	177	62	140.9	267.6	15	395.2	6980	12.4	193000	139
39	75	27.51	130	79	113	66	50.4	188.8	20	356.2	9160	14.3	241000	135
40	67	31.20	155	92	106	63	83.4	186.1	20	272.8	6040	15.4	368000	141

41	70	32.47	150	58	146	43	116.1	212.7	17	339.4	6980	12.0	290000	145
42	59	29.54	120	6	30	62	39.7	99.6	5	288.9	5120	15.1	281000	134
43	46	27.64	120	5	159	62	74.8	235.7	10	308.6	6090	12.1	238000	141
44	51	34.12	140	16	94	58	428.8	238.1	9	346.0	8980	14.4	278000	138
45	50	29.01	140	8	263	61	103.7	186.3	2	200.5	6080	14.7	425000	139
46	52	26.57	140	22	96	60	314.8	219.1	10	233.3	8980	14.9	360000	141
47	19	24.78	120	6	117	56	143.5	194.1	10	326.1	6080	13.3	328000	145
48	57	29.78	140	3	178	52	139.7	270.8	2	283.4	6690	15.1	253000	134
49	56	29.05	130	12	178	61	381.1	315.0	20	407.4	7070	16.1	274000	141
50	56	27.08	135	9	158	63	88.8	238.9	8	301.8	7320	15.1	425000	140
51	51	30.12	130	6	216	61	66.2	290.6	10	394.8	6540	14.6	188000	137
52	77	28.55	150	46	170	62	76.8	269.0	11	407.4	9160	17.1	266000	133
53	57	31.25	125	10	255	58	120.6	337.2	20	360.7	6040	14.6	220000	135
54	55	30.46	135	10	263	63	238.8	193.0	2	433.4	6980	15.1	300000	140
55	49	30.35	150	5	110	63	141.9	201.9	5	339.4	7120	12.7	425000	139
56	66	25.06	140	6	77	63	76.2	155.0	13	545.6	13300	13.3	208000	142
57	58	38.74	150	105	158	72	155.8	191.8	12	383.4	9540	13.2	262000	140
58	59	31.89	145	37	216	61	118.4	292.0	25	346.0	6010	16.1	152000	137
59	38	26.45	120	42	170	57	154.9	248.6	4	288.9	9540	15.9	274000	133
60	35	31.89	145	5	158	59	80.7	292.0	10	433.4	4190	14.3	145000	135
61	61	30.43	170	3	166	57	374.0	297.6	5	283.4	5460	13.3	264000	140

62	59	33.41	120	37	216	55	144.8	219.5	5	312.6	10050	13.2	152000	135
63	66	28.69	130	29	198	57	105.1	276.3	35	265.4	9540	16.1	274000	139
64	61	18.97	120	10	216	52	74.0	281.0	15	433.6	4190	12.7	145000	142
65	62	30.72	140	5	103	67	146.4	199.3	2	312.6	6150	15.0	270000	144
66	62	32.39	140	3	65	62	121.3	151.6	15	272.8	6010	12.9	190000	139
67	64	27.99	135	27	216	61	80.7	155.0	10	319.0	13300	15.3	166000	142
68	65	26.79	150	5	211	59	129.7	316.0	5	255.9	7220	12.9	211000	142
69	43	30.07	130	6	43	58	356.6	172.5	5	295.3	5620	18.0	242000	140
70	49	25.24	160	14	164	65	61.8	241.0	30	433.6	5680	13.3	263000	140
71	33	23.94	110	11	181	81	80.7	250.6	6	262.8	5860	12.9	237000	137
72	57	29.41	160	22	181	58	66.8	252.4	10	299.7	5410	14.4	170000	133
73	57	23.53	120	16	192	82	76.7	288.9	10	373.2	7490	14.9	263000	135
74	42	24.61	100	10	187	68	22.8	259.4	7	284.8	5540	12.7	399000	140
75	62	30.85	150	6	114	61	231.6	221.4	20	413.3	6860	11.0	310000	135
76	40	27.48	110	22	144	52	131.5	221.9	18	259.1	3860	12.6	212000	133
77	57	36.83	150	6	148	64	66.5	225.6	8	283.4	6520	13.7	342000	135
78	33	25.81	120	75	196	62	77.2	273.9	15	302.4	9020	13.4	225000	140
79	70	27.92	130	29	181	70	85.3	261.2	10	462.2	6680	13.9	297000	135
80	54	37.65	160	46	155	63	71.4	231.8	5	355.5	7030	11.8	262000	144
81	57	26.22	120	17	175	64	155.4	270.3	30	283.4	7940	14.7	472000	139
82	53	26.70	140	36	98	71	73.9	183.4	20	316.3	4540	11.8	368000	138

83	61	32.10	150	18	128	63	80.6	207.0	12	247.0	5650	13.4	274000	142
84	86	23.15	140	60	174	58	87.0	210.0	27	489.4	8520	15.2	270000	139
85	90	22.89	130	17	133	55	151.0	158.0	15	346.0	4120	13.2	251000	140
86	57	31.90	140	20	174	72	70.9	259.3	8	249.9	4580	12.4	307000	141
87	57	34.20	130	7	133	61	355.6	265.0	3	211.5	5370	12.4	234000	142
88	62	20.55	150	9	134	82	128.8	241.5	5	323.6	8590	12.1	86000	141
89	56	30.71	110	12	178	61	381.1	315.0	30	283.4	7070	11.4	262000	143
90	54	27.20	140	37	201	61	83.7	278.3	10	330.3	7440	13.4	240000	141
91	61	30.12	140	141	143	60	114.0	211.0	7	420.0	11480	14.9	319000	143
92	64	26.12	130	15	93	61	50.0	155.0	16	420.0	4700	13.4	266000	139
93	50	27.27	130	53	181	66	151.5	277.0	15	331.2	6490	14.6	466000	140
94	54	25.95	120	12	172	55	195.8	265.4	5	216.0	5610	12.7	206000	141
95	54	25.51	125	10	182	66	214.7	291.5	15	392.1	6240	14.5	186000	143
96	62	27.40	140	32	90	46	298.8	196.1	5	233.2	7830	14.5	316000	143
97	65	27.82	130	5	158	61	188.6	257.4	20	244.1	6060	14.0	205000	142
98	57	35.49	110	11	168	57	326.2	290.2	15	338.1	11480	15.3	152000	139
99	56	39.18	140	6	178	61	212.2	281.4	20	331.2	4200	14.2	163000	141
100	75	29.40	150	21	185	61	45.3	254.9	32	373.2	6220	15.2	244000	139
101	70	26.64	130	66	162	57	80.7	235.5	30	283.4	4560	14.9	219000	141
102	62	34.42	140	7	122	42	109.4	186.2	25	331.2	11480	14.1	187000	140
103	59	24.22	120	13	126	68	65.5	206.6	10	309.3	6740	14.9	410000	142

104	65	26.99	135	12	184	52	135.7	247.3	15	334.2	7320	12.0	344000	141
105	57	31.63	120	26	144	67	157.8	242.3	10	392.1	5460	12.2	334000	140
106	67	28.41	170	30	176	58	46.4	243.6	5	423.9	6010	14.6	236000	145
107	71	27.99	110	66	47	59	46.6	115.5	20	271.1	5180	12.2	247000	141
108	59	32.83	130	37	174	62	98.0	256.0	2	392.1	8420	16.7	289000	140
109	56	35.40	120	42	95	61	78.8	171.3	10	421.1	8180	15.1	243000	140
110	61	29.73	125	4	195	62	95.9	276.1	10	233.2	5780	14.6	164000	142
111	64	29.63	130	3	169	61	196.7	269.3	15	364.2	12990	13.5	261000	140
112	58	29.41	140	11	140	63	81.0	219.0	2	205.7	5550	16.0	263000	140
113	42	34.22	180	21	169	63	504.2	206.7	8	321.2	11370	14.9	310000	142
114	63	33.22	140	3	135	64	238.2	246.6	9	383.4	4690	13.1	280000	142
115	66	28.73	170	3	116	66	137.9	209.2	10	238.0	8180	15.4	310000	140
116	78	32.63	130	34	135	61	203.2	236.2	10	255.9	10020	13.3	309000	139
117	56	39.18	135	6	178	61	212.2	281.4	20	331.2	4200	15.7	263000	144
118	65	36.45	140	21	101	65	155.3	196.7	25	365.6	5380	15.7	268000	139
119	53	23.53	130	13	225	61	126.5	311.5	20	348.7	6340	14.6	215000	140
120	67	28.73	130	3	97	65	131.7	188.7	3	289.3	10020	13.3	187000	139
121	67	34.89	150	29	89	66	42.7	163.7	15	339.0	6820	14.9	202000	141
122	67	26.46	120	10	71	44	36.5	122.4	5	331.2	5230	13.3	210000	139
123	75	29.17	130	5	225	63	155.3	267.5	25	365.6	7020	14.2	264000	142
124	63	35.30	140	11	138	66	40.3	212.4	12	252.9	4080	15.5	256000	138

125	79	30.01	130	5	192	66	93.4	277.0	5	259.1	11410	12.6	142000	142
126	77	29.10	140	5	147	52	136.8	226.8	20	302.4	5480	15.5	315000	144
127	57	26.47	130	3	127	66	98.3	212.8	3	253.4	6020	11.2	229000	141
128	60	31.24	120	7	171	61	212.9	274.1	10	325.6	7970	13.5	271000	138
129	52	23.62	120	86	148	61	86.5	226.0	35	470.7	4810	15.4	376000	142
130	55	39.46	135	13	65	62	106.5	148.4	3	300.4	6100	12.9	267000	138
131	65	32.93	125	46	91	62	45.5	161.3	10	302.4	5540	14.6	246000	140
132	56	28.78	130	3	195	62	104.7	278.4	5	393.6	5320	15.1	397000	138
133	77	29.24	165	3	179	56	269.2	288.4	10	572.5	7250	11.1	158000	142
134	75	34.67	130	47	41	56	131.9	123.3	5	340.7	7970	15.0	185000	141
135	70	25.20	130	21	65	58	167.5	156.4	12	374.4	7630	15.4	323000	139
136	71	40.90	145	99	177	58	239.2	271.9	20	458.3	6910	13.8	160000	141
137	64	25.81	140	5	149	57	346.0	275.2	5	340.7	7120	14.7	225000	136
138	72	31.41	190	18	198	65	134.5	290.2	15	413.3	7370	15.3	197000	141
139	56	24.80	125	5	143	65	122.9	232.6	2	403.1	6250	14.3	263000	140
140	56	23.40	140	5	100	66	56.7	177.1	15	374.4	7370	15.4	254000	135
141	66	27.34	130	16	119	66	156.7	216.6	12	302.4	6820	14.5	216000	133
142	44	19.92	120	6	154	62	57.5	227.7	6	294.6	6910	15.2	322000	135
143	57	27.77	145	20	82	62	68.1	157.1	8	272.2	7510	14.9	397000	140
144	71	32.37	150	21	157	57	119.9	237.7	12	378.5	9480	11.4	247000	146
145	43	22.77	110	23	169	65	212.7	276.8	15	216.0	7560	12.8	299000	136

146		40	20.45	120	12	225	57	212.7	196.9	8	446.4	5320	12.9	321000	135
147		60	24.68	140	57	225	65	63.5	206.0	5	220.4	4610	14.7	304000	134
148		62	26.15	140	5	65	57	41.8	130.7	25	446.4	9480	11.4	212000	137
149		68	25.61	130	29	285	56	114.8	364.3	5	403.1	5030	13.7	199000	141
150		73	28.73	140	4	120	65	82.4	201.1	25	446.4	7200	12.8	189000	143
151		73	25.28	150	12	145	62	45.6	216.6	40	393.2	5360	13.5	212000	142
152		38	14.18	110	3	225	55	82.4	224.5	8	570.5	5360	14.7	304000	140
153		62	28.78	140	40	200	62	249.5	311.5	10	378.5	6470	14.8	270000	139
Total	Median	58.00	28.7300	130.00	17.00	161.00	61.00	114.000	236.200	10.00	331.200	6430.00	14.300	263000.00	139.80
	Minimum	19	14.18	100	3	30	42	22.8	99.6	2	200.5	3860	11.0	86000	131
	Maximum	90	47.47	190	143	285	82	504.2	364.3	55	572.5	13300	18.0	491000	146
	Mean	57.88	28.7927	134.02	30.14	155.19	61.41	137.171	234.676	13.43	337.078	6928.76	14.110	269385.62	139.35
	Std. Deviation	12.296	4.86619	15.369	31.502	51.389	5.998	89.9492	52.5017	9.842	81.1751	2000.160	1.3451	77735.476	3.005

	AST (U/L)	ALT (U/L)	Creatinine (mg/dL)	Urea (mg/dL)	Uric acid (mg/dL)	Serum calcium (mg/dL)	Magnesium (mg/dL)	Blood glucose	Glycated Hb	eGFR	CK (UI/mL)	Apolipoprotein B	Waist_circumference	TSH (mUI/mL)	GGT
1	26.56	25.32	0.89	26.1	3.8	9.1	2.3	107	5.7	70.40	211	169	99	1.69	99
2	16.57	24.22	0.84	58.6	4.5	9.8	1.8	145	6.1	95.07	78	139	112	1.39	112
3	14.33	25.31	0.85	26.0	6.7	10.6	2.1	93	5.4	104.31	66	152	102	1.52	102
4	10.90	17.10	0.68	33.6	5.8	10.2	2.0	89	5.0	65.66	142	138	86	1.38	86
5	25.71	25.62	0.91	33.0	6.3	9.0	2.2	118	5.9	89.97	72	183	93	1.83	93
6	24.46	18.07	0.65	41.3	4.5	9.1	2.3	105	5.7	70.40	227	152	66	1.39	66
7	24.46	23.27	0.85	33.1	5.8	9.1	1.8	108	6.1	95.07	57	138	72	1.52	72
8	16.55	18.61	1.17	26.3	5.1	9.8	2.1	107	5.4	104.31	78	183	78	1.38	78
9	19.92	16.11	0.65	26.1	3.8	10.6	2.2	87	7.5	132.37	66	110	68	1.10	68
10	29.86	21.45	0.85	58.6	4.5	9.8	2.3	124	5.6	113.42	142	108	102	1.03	89
11	23.01	23.27	1.02	26.0	3.5	9.3	1.8	77	5.0	87.97	72	121	86	1.18	77
12	9.83	11.84	0.89	21.4	3.8	9.1	1.8	84	5.9	113.42	227	104	76	1.04	76
13	29.86	21.54	0.97	26.1	4.5	10.9	2.3	145	5.3	88.30	57	183	78	1.71	78
14	23.01	28.56	1.02	58.6	6.7	10.5	1.7	93	7.5	88.30	210	108	98	1.08	98
15	23.27	14.17	0.76	26.0	4.5	9.2	2.1	71	5.6	76.10	66	121	89	1.21	89
16	27.99	21.45	1.18	33.9	3.8	10.9	1.7	83	5.0	107.29	155	155	77	1.28	77
17	20.43	27.53	1.11	33.5	4.5	10.5	1.9	90	5.3	84.11	165	126	109	0.26	109
18	29.79	45.55	1.05	34.8	6.7	8.8	2.1	101	7.5	88.31	91	155	118	1.55	118

19	87.03	159.87	0.77	22.5	6.1	8.5	1.8	80	5.6	88.30	87	103	95	1.03	95
20	21.88	14.47	0.82	40.1	5.2	9.7	2.3	107	5.0	74.88	155	118	98	1.18	98
21	17.92	15.36	0.91	44.9	6.8	10.2	2.4	145	6.0	74.65	142	137	94	1.37	94
22	15.99	22.49	0.91	50.9	2.7	8.8	1.7	93	5.0	83.92	87	128	98	1.28	98
23	23.80	27.92	0.74	58.6	7.3	10.6	1.9	92	5.2	74.57	142	128	121	1.28	82
24	30.35	38.92	2.18	19.6	4.5	9.4	1.9	104	5.5	70.04	87	171	126	1.71	126
25	34.56	23.21	0.99	26.0	6.6	9.4	1.6	101	7.5	69.54	203	117	108	1.17	108
26	16.72	30.26	1.54	40.0	3.3	10.4	2.1	100	7.5	91.84	66	155	122	1.64	122
27	35.28	47.42	0.76	34.1	4.2	10.1	1.9	102	5.6	84.86	155	127	104	1.27	104
28	343.00	360.20	0.83	26.1	7.3	8.8	2.3	96	5.0	54.58	165	57	92	0.57	92
29	33.58	27.04	0.90	58.6	5.7	10.3	2.1	70	7.9	43.18	68	121	96	1.21	96
30	14.24	30.87	1.29	26.0	5.3	9.7	2.1	103	5.9	82.32	66	57	99	0.95	99
31	27.02	31.60	0.81	17.7	4.5	9.4	1.6	102	5.4	113.20	142	145	98	1.45	98
32	21.17	24.83	0.94	36.0	3.7	10.4	2.1	79	6.9	72.49	87	124	88	1.24	88
33	24.99	36.93	0.89	32.8	4.6	10.1	1.9	110	6.5	60.71	142	164	76	1.64	76
34	24.46	45.54	1.29	26.9	5.0	9.7	1.7	107	6.1	78.39	87	112	78	1.12	78
35	19.20	21.90	0.84	38.2	6.6	10.3	1.9	99	5.4	63.22	130	71	98	0.71	98
36	29.22	55.70	1.14	22.4	4.8	9.3	2.1	104	6.9	71.94	142	128	104	1.28	104
37	20.25	20.53	0.92	58.6	7.3	9.1	1.9	151	5.8	71.09	87	95	85	0.95	85
38	17.50	23.27	0.80	41.0	4.4	10.7	2.0	120	4.8	92.53	149	127	94	1.27	94
39	19.26	28.54	0.85	30.3	8.4	8.3	1.9	102	6.3	75.62	87	83	86	0.83	86

40	19.59	13.72	0.97	21.9	3.7	9.5	1.7	95	8.2	71.09	130	20	100	0.20	100
41	28.45	38.35	0.95	20.9	4.1	10.2	2.4	86	5.8	83.00	142	114	110	1.14	110
42	27.12	40.03	0.80	50.4	4.8	9.1	2.2	83	4.8	67.02	87	47	102	0.47	102
43	15.86	20.25	0.72	37.4	7.9	10.4	2.2	112	6.3	60.43	220	112	89	1.12	89
44	38.68	68.91	0.70	29.9	4.1	10.6	2.1	95	9.5	60.68	165	190	104	1.90	104
45	24.79	52.93	0.92	26.7	3.7	10.1	2.1	65	4.8	97.78	438	76	108	0.76	108
46	21.59	29.43	0.66	40.8	4.2	10.1	1.9	81	6.3	66.33	87	165	95	1.65	95
47	16.18	24.52	0.74	32.4	7.9	9.0	2.1	77	9.5	109.27	130	92	82	0.92	82
48	27.95	68.80	1.09	22.5	4.2	10.5	2.5	106	7.2	96.64	142	131	106	1.31	106
49	35.94	33.95	0.83	34.7	6.5	10.2	1.9	102	4.8	111.16	87	204	125	2.04	125
50	50.83	47.54	1.08	34.5	5.7	9.2	1.8	87	5.9	66.33	165	109	94	1.09	94
51	23.32	25.65	0.74	44.0	8.0	10.7	1.8	126	5.4	74.95	250	161	98	1.61	98
52	25.99	30.59	0.73	28.8	4.9	8.6	1.8	89	4.8	78.83	72	64	106	0.64	106
53	26.53	37.83	0.75	29.1	7.3	9.9	1.8	115	5.9	76.32	65	138	104	1.38	104
54	21.23	35.03	1.22	23.1	3.6	9.4	1.8	81	5.4	93.80	72	105	121	1.05	121
55	17.81	24.13	1.13	38.9	8.4	9.9	1.7	114	6.3	79.44	72	117	118	1.17	118
56	16.62	21.40	0.94	35.0	3.1	10.0	1.8	148	9.5	89.10	83	105	100	1.05	100
57	83.24	87.98	0.99	47.5	7.2	9.4	1.9	172	7.2	66.33	165	30	121	0.30	121
58	12.76	24.14	0.80	37.6	7.9	8.5	2.5	106	5.6	75.90	250	128	104	1.28	104
59	24.01	36.13	1.02	40.9	2.5	10.3	1.7	121	8.1	70.36	78	157	82	1.57	82
60	24.46	45.54	1.29	48.4	7.9	10.2	1.9	128	6.9	83.60	211	71	121	0.95	125

61	24.53	24.86	0.72	37.8	3.2	9.6	2.2	86	5.4	80.70	78	162	92	1.62	92
62	178.30	195.47	0.99	17.5	3.9	10.5	2.2	93	7.2	92.81	80	136	115	1.38	115
63	72.30	115.98	0.77	48.4	6.6	10.2	1.9	151	5.6	93.80	114	132	97	1.32	97
64	10.74	18.90	0.69	44.7	5.2	9.1	2.5	94	8.1	90.38	301	106	61	1.06	61
65	19.56	35.76	0.83	30.9	9.4	9.2	2.5	121	6.9	84.50	78	101	115	1.01	115
66	15.71	17.86	0.87	30.6	6.9	10.6	2.2	108	5.4	94.56	250	72	105	0.72	105
67	16.86	17.02	0.99	41.1	4.3	10.1	1.8	103	6.9	93.96	117	93	96	0.93	96
68	19.88	21.21	1.22	29.2	5.8	10.1	2.5	90	5.8	94.30	124	147	102	1.47	102
69	52.27	99.88	1.13	42.5	4.5	8.4	2.1	129	7.2	71.39	80	101	108	1.01	108
70	18.76	20.53	0.88	37.0	6.6	10.5	2.6	151	6.3	84.50	114	94	80	0.94	80
71	12.83	17.52	0.85	30.9	7.2	10.3	1.7	117	5.8	61.83	301	121	90	1.21	90
72	18.17	12.85	1.12	31.5	7.8	10.6	2.5	151	7.1	79.17	117	104	89	1.01	89
73	19.45	7.13	0.85	31.4	5.5	9.2	2.4	85	5.1	77.15	124	138	76	1.38	76
74	15.53	9.40	0.92	19.9	3.5	9.5	1.9	89	6.1	90.02	301	106	86	1.06	86
75	32.73	19.22	0.94	38.7	5.2	10.2	1.8	94	4.8	54.49	117	153	104	1.53	104
76	19.57	14.79	0.86	30.4	6.4	10.1	2.2	92	7.8	76.06	70	112	88	1.12	88
77	19.10	34.36	0.78	26.6	5.8	10.3	1.9	79	6.1	103.80	301	117	126	1.17	126
78	16.84	15.52	1.08	30.1	6.9	10.3	1.9	90	5.0	86.54	117	105	79	1.05	79
79	12.48	20.45	0.75	23.9	5.2	8.9	1.9	95	5.4	84.50	98	136	98	1.36	98
80	88.02	82.39	0.99	35.3	3.5	8.4	2.2	103	5.3	100.20	152	103	125	1.03	125
81	24.46		0.77	16.4	4.5	9.0	2.0	88	6.1	67.39	118	146	90	1.46	90

82	26.52	21.47	0.95	22.6	4.4	8.1	2.5	111	6.1	80.76	355	67	94	0.67	94
83	13.48	13.35	0.55	29.0	6.1	9.0	1.6	131	7.8	85.99	133	93	102	0.93	102
84	13.00	22.00	0.65	66.1	4.5	10.4	1.9	109	7.1	85.71	72	103	88	1.53	88
85	16.00	16.00	0.72	28.2	4.8	10.1	2.0	100	5.3	68.38	90	146	82	2.04	82
86	22.82	19.70	0.64	40.4	5.3	9.6	2.5	91	5.1	112.47	98	88	92	0.88	92
87	39.10	46.74	1.10	41.6	5.3	9.4	1.9	89	5.0	80.39	152	199	102	1.99	102
88	19.22	27.23	0.95	45.9	4.2	9.2	2.2	93	8.3	82.13	118	108	62	1.08	62
89	35.94	33.95	0.83	52.1	3.5	9.9	1.9	94	5.7	99.06	355	204	98	2.04	98
90	12.12	18.99	0.64	39.3	5.3	10.0	1.9	105	5.3	74.12	72	133	78	1.33	78
91	23.00	14.00	0.95	36.6	5.3	9.1	1.9	94	5.5	64.19	55	114	97	1.14	97
92	13.00	15.00	0.72	28.8	4.9	10.1	2.5	89	5.0	78.83	72	74	106	0.74	106
93	19.08	10.69	0.99	18.1	3.6	10.6	1.7	99	9.3	101.17	117	133	82	1.33	82
94	19.35	40.62	0.87	55.0	5.7	8.9	2.2	128	7.4	67.90	55	162	92	1.62	92
95	28.91	16.48	1.03	38.0	4.4	9.3	2.0	136	6.3	103.80	117	185	98	1.85	98
96	12.85	34.14	1.10	24.2	4.8	10.2	1.9	83	6.4	66.44	124	132	105	1.32	105
97	27.97	19.85	0.66	31.3	6.8	9.2	1.7	95	5.1	97.84	98	137	89	1.37	89
98	19.24	23.98	0.72	26.9	5.8	9.6	2.5	77	5.0	61.58	117	212	122	2.12	122
99	82.29	155.13	1.09	34.5	6.7	8.9	1.6	158	9.3	73.61	124	172	132	1.72	132
100	20.65	29.98	0.83	30.6	3.7	10.2	2.2	80	5.0	92.70	55	112	102	1.12	102
101	15.00	12.54	0.85	34.9	6.8	10.6	2.2	108	5.9	92.96	98	107	97	1.07	97
102	16.59	30.49	1.30	27.4	8.5	10.5	2.1	116	6.1	75.48	152	102	125	1.02	125

103	12.80	9.78	0.95	32.0	7.0	9.7	1.8	82	6.1	68.98	118	87	77	0.87	77
104	27.28	31.87	1.34	34.6	3.5	8.6	2.2	86	5.9	69.42	355	150	102	1.50	102
105	21.77	20.82	0.99	66.1	7.2	8.9	2.2	113	7.8	58.48	61	75	111	0.75	111
106	35.16	32.50	1.20	23.4	4.6	9.6	2.2	84	5.6	65.56	402	96	105	0.96	105
107	31.41	34.04	1.10	34.2	5.7	8.6	1.7	82	6.4	55.20	83	141	97	1.41	97
108	17.55	16.33	0.85	42.8	4.6	9.6	2.2	89	5.4	63.25	152	145	110	1.45	110
109	24.03	28.76	1.20	31.0	9.3	9.7	1.9	98	5.6	62.20	402	80	115	0.80	115
110	36.18	49.31	0.97	28.3	7.4	9.5	1.9	98	5.0	67.18	93	139	98	1.39	98
111	31.26	34.27	1.15	43.2	5.9	9.9	2.1	92	5.5	95.37	136	159	115	1.59	115
112	14.87	21.79	0.97	66.1	4.5	9.4	1.7	153	5.9	54.58	174	98	98	0.98	98
113	13.96	43.54	0.84	40.8	5.5	10.2	2.0	88	5.1	63.04	141	88	103	0.88	103
114	45.98	79.61	1.06	20.5	5.9	10.1	1.7	87	5.4	66.88	402	125	118	1.25	118
115	31.24	43.43	0.86	25.1	5.3	9.9	1.9	91	5.6	64.38	165	114	103	1.14	103
116	24.46	23.21	0.99	36.8	7.7	9.4	1.8	105	5.4	107.99	152	117	107	1.17	107
117	82.29	155.13	1.09	25.6	4.7	9.9	1.7	104	5.8	74.32	123	172	127	1.72	127
118	24.53	9.95	0.98	33.6	4.6	8.6	1.9	178	5.9	90.36	86	103	122	1.03	122
119	12.82	7.28	1.18	26.1	7.3	8.8	2.3	96	5.0	54.58	165	156	87	1.56	87
120	35.39	16.53	1.22	27.4	8.5	10.5	2.1	116	6.1	75.48	152	102	110	1.02	110
121	25.72	19.63	1.15	55.6	5.1	9.2	1.7	107	5.6	60.53	53	67	122	1.11	122
122	45.54	56.21	1.20	36.6	4.8	10.3	1.9	85	5.1	52.61	56	68	86	0.68	86
123	24.81	16.87	1.25	19.9	5.9	10.3	2.3	79	5.0	60.97	136	140	98	1.40	98

124	12.37	30.36	0.97	41.7	5.7	10.1	1.7	91	5.2	49.19	174	110	123	1.10	123
125	23.24	18.14	0.83	31.1	5.5	8.6	1.8	122	5.0	62.20	86	138	101	1.38	101
126	29.52	28.23	0.97	28.7	4.3	9.6	1.9	88	7.5	42.05	51	117	98	1.17	98
127	35.14	89.67	0.52	32.4	6.7	9.2	1.7	92	5.4	82.74	86	102	96	1.02	96
128	25.84	22.58	0.85	42.4	4.7	9.1	2.3	81	5.8	67.07	136	156	95	1.56	95
129	15.39	12.05	0.78	39.0	4.5	8.9	1.7	96	5.4	56.33	174	106	79	1.06	79
130	26.15	18.91	0.87	26.4	4.1	10.3	2.3	98	5.6	118.37	141	89	135	0.89	135
131	14.33	16.44	0.81	40.4	6.2	9.6	2.3	81	5.9	74.47	164	67	117	0.67	117
132	21.67	20.69	0.78	50.2	3.1	9.9	1.8	84	4.8	87.40	34	121	96	1.21	96
133	23.09	16.51	1.19	44.9	7.2	9.4	1.9	105	5.3	97.16	164	137	106	1.37	106
134	11.93	15.17	1.57	43.9	7.2	8.6	2.2	91	7.5	93.27	51	106	113	1.02	113
135	30.26	38.94	0.76	31.0	5.2	9.3	2.5	87	5.4	84.98	86	89	101	1.56	101
136	45.54	45.54	0.95	50.2	5.5	9.6	2.0	69	5.3	44.00	159	198	117	1.98	117
137	16.90	18.01	0.75	37.2	5.4	9.1	2.2	82	8.1	42.49	136	137	90	1.37	90
138	24.89	17.10	1.06	27.2	5.3	9.4	1.7	129	7.5	79.48	174	123	122	1.23	122
139	22.68	21.05	0.98	37.1	4.7	9.6	1.9	144	5.1	60.26	185	105	94	1.05	94
140	17.59	13.32	0.90	24.6	5.2	10.6	2.0	167	7.8	84.24	47	100	86	1.00	86
141	60.43	38.13	0.66	30.8	6.1	9.1	2.4	89	5.8	69.77	152	97	86	0.97	86
142	24.32	11.73	0.79	37.1	4.3	9.5	1.8	86	5.3	85.83	53	88	56	0.88	56
143	25.59	20.47	0.92	19.2	2.7	8.8	1.7	144	9.9	71.48	56	63	88	0.63	88
144	13.60	13.49	1.08	40.4	4.6	10.0	2.2	86	7.5	92.05	371	106	125	1.06	125

145		9.85	13.42	1.11	31.3	4.7	8.6	1.7	80	5.4	91.04	112	82	122	0.82	90
146		15.11	15.28	0.77	33.4	4.0	9.9	2.2	91	8.0	69.12	113	65	94	0.65	122
147		19.95	29.41	0.83	40.1	7.2	9.5	1.8	86	5.6	51.60	164	76	88	0.76	88
148		24.54	23.80	1.00	28.5	3.9	10.0	1.6	92	7.5	60.77	34	71	92	0.71	92
149		31.73	24.82	0.84	30.0	4.8	10.0	1.7	84	5.4	140.14	164	155	111	1.55	111
150		28.95	23.20	1.01	20.7	4.4	8.8	2.0	91	8.0	76.64	51	132	100	1.32	100
151		22.56	15.40	0.75	26.4	6.1	9.9	1.7	75	5.2	67.65	96	117	98	1.17	98
152		25.20	6.80	0.83	31.5	4.3	9.1	2.3	85	5.4	71.41	149	71	43	0.71	43
153		22.61	27.10	0.87	25.0	6.5	8.3	3.2	119	5.7	73.45	149	177	96	1.77	96
Total	Median	23.0900	23.5350	0.9100	33.000	5.200	9.600	1.940	95.20	5.800	76.6400	118.00	117.00	98.00	1.1700	98.00
	Minimum	9.83	6.80	0.52	16.4	2.5	8.1	1.6	65	4.8	42.05	34	20	43	0.20	43
	Maximum	343.00	360.20	2.18	66.1	9.4	10.9	3.2	178	9.9	140.14	438	212	135	2.12	135
	Mean	28.7655	34.0971	0.9360	34.631	5.412	9.650	2.008	101.95	6.123	79.2848	136.40	119.87	98.73	1.2048	98.33
	Std. Deviation	31.82244	38.93993	0.21038	10.6042	1.4578	0.6587	0.2710	22.072	1.1517	17.43529	80.514	36.013	15.872	0.36328	15.955

a. Limited to first 300 cases.

SPEARMAN AND PEARSON CORRELATIONS

Table S4. Pearson correlation analysis between Lp(a), body mass index, lipid profile, and cardiovascular disease

		Lp(a) (mg/dL)	BMI (kg/m²)	Cardiovascular diseases	LDL-C (mg/dL)	HDL-C (mg/dL)	Triglycerides (mg/dL)	Total cholesterol (mg/dL)
Lp(a) (mg/dL or nmol/L)	Pearson Correlation	1	-.041	-.125	.075	-.034	-.144	.040
	Sig. (2-tailed)		.619	.125	.356	.678	.077	.621
	N	153	153	153	153	153	153	153
BMI (kg/m²)	Pearson Correlation	-.041	1	.293**	-.121	-.084	.244**	.001
	Sig. (2-tailed)	.619		<.001	.135	.303	.002	.990
	N	153	153	153	153	153	153	153
Cardiovascular diseases	Pearson Correlation	-.125	.293**	1	-.159*	-.148	.086	-.179*
	Sig. (2-tailed)	.125	<.001		.050	.067	.291	.027
	N	153	153	153	153	153	153	153
LDL-C (mg/dL)	Pearson Correlation	.075	-.121	-.159*	1	-.060	.047	.776**
	Sig. (2-tailed)	.356	.135	.050		.462	.566	<.001
	N	153	153	153	153	153	153	153
HDL-C (mg/dL)	Pearson Correlation	-.034	-.084	-.148	-.060	1	-.225**	.018
	Sig. (2-tailed)	.678	.303	.067	.462		.005	.823
	N	153	153	153	153	153	153	153
Triglycerides (mg/dL)	Pearson Correlation	-.144	.244**	.086	.047	-.225**	1	.232**
	Sig. (2-tailed)	.077	.002	.291	.566	.005		.004
	N	153	153	153	153	153	153	153
Total cholesterol (mg/dL)	Pearson Correlation	.040	.001	-.179*	.776**	.018	.232**	1
	Sig. (2-tailed)	.621	.990	.027	<.001	.823	.004	
	N	153	153	153	153	153	153	153

**. Correlation is significant at the 0.01 level (2-tailed).

*. Correlation is significant at the 0.05 level (2-tailed).

Table S5. Spearman correlation analysis between Lp(a), body mass index, lipid profile, and cardiovascular disease

			Lp(a) (mg/dL)	BMI (kg/m²)	Cardiovascular diseases	LDL-C (mg/dL)	HDL-C (mg/dL)	Triglycerides (mg/dL)	Total cholesterol (mg/dL)
Spearman's rho	Lp(a) (mg/dL)	Correlation Coefficient	1.000	-.029	-.057	.062	-.051	-.109	-.002
		Sig. (2-tailed)	.	.718	.487	.450	.534	.178	.981
		N	153	153	153	153	153	153	153

	BMI (kg/m²)	Correlation Coefficient	-.029	1.000	.341**	-.137	-.039	.256**	-.024
		Sig. (2-tailed)	.718	.	<.001	.092	.633	.001	.765
		N	153	153	153	153	153	153	153
	Cardiovascular diseases	Correlation Coefficient	-.057	.341**	1.000	-.151	-.124	.059	-.179*
		Sig. (2-tailed)	.487	<.001	.	.062	.128	.468	.027
		N	153	153	153	153	153	153	153
	LDL-C (mg/dL)	Correlation Coefficient	.062	-.137	-.151	1.000	-.178*	.086	.740**
		Sig. (2-tailed)	.450	.092	.062	.	.028	.291	<.001
		N	153	153	153	153	153	153	153
	HDL-C (mg/dL)	Correlation Coefficient	-.051	-.039	-.124	-.178*	1.000	-.233**	-.098
		Sig. (2-tailed)	.534	.633	.128	.028	.	.004	.226
		N	153	153	153	153	153	153	153
	Triglycerides (mg/dL)	Correlation Coefficient	-.109	.256**	.059	.086	-.233**	1.000	.258**
		Sig. (2-tailed)	.178	.001	.468	.291	.004	.	.001
		N	153	153	153	153	153	153	153
	Total cholesterol (mg/dL)	Correlation Coefficient	-.002	-.024	-.179*	.740**	-.098	.258**	1.000
		Sig. (2-tailed)	.981	.765	.027	<.001	.226	.001	.
		N	153	153	153	153	153	153	153

**. Correlation is significant at the 0.01 level (2-tailed).

*. Correlation is significant at the 0.05 level (2-tailed).

REGRESSION ANALYSIS

Table S6. Independent variables included in the regression model with Lp(a) as the dependent variable

Variables Entered/Removed[a]

Model	Variables Entered	Variables Removed	Method
1	Male, Triglycerides (mg/dL), Diabetes mellitus (Yes/No), BMI (kg/m²), LDL-C (mg/dL)[b]	.	Enter

a. Dependent Variable: Lp(a) (mg/dL or nmol/L)

b. All requested variables entered.

Table S7. Summary of multivariable linear regression model for Lp(a) levels

Model Summary[b]

Model	R	R Square	Adjusted R Square	Std. Error of the Estimate
1	.214[a]	.046	.013	31.294

a. Predictors: (Constant), Male, Triglycerides (mg/dL), Diabetes mellitus (Yes/No), BMI (kg/m²), LDL-C (mg/dL)
b. Dependent Variable: Lp(a) (mg/dL or nmol/L)

Table S8. ANOVA results for predictors of Lp(a) in the regression model

ANOVA[a]

Model		Sum of Squares	df	Mean Square	F	Sig.
1	Regression	6878.048	5	1375.610	1.405	.226[b]
	Residual	143960.069	147	979.320		
	Total	150838.118	152			

a. Dependent Variable: Lp(a) (mg/dL or nmol/L)
b. Predictors: (Constant), Male, Triglycerides (mg/dL), Diabetes mellitus (Yes/No), BMI (kg/m²), LDL-C (mg/dL)

Table S9. Effects of demographic and metabolic variables on Lp(a) in the regression model

Coefficients[a]

Model		Unstandardized Coefficients		Standardized Coefficients		
		B	Std. Error	Beta	t	Sig.
1	(Constant)	31.960	18.191		1.757	.081
	LDL-C (mg/dL)	.036	.052	.059	.700	.485
	Triglycerides (mg/dL)	-.049	.029	-.141	-1.683	.095
	BMI (kg/m²)	.070	.549	.011	.128	.898
	Diabetes mellitus (Yes/No)	5.136	7.235	.058	.710	.479
	Male	-8.178	5.329	-.129	-1.535	.127

a. Dependent Variable: Lp(a) (mg/dL or nmol/L)

Table S10. Distribution of predicted values and residuals in the regression model for Lp(a)

Residuals Statistics[a]

	Minimum	Maximum	Mean	Std. Deviation	N
Predicted Value	7.54	42.66	30.14	6.727	153
Residual	-37.492	115.665	.000	30.775	153
Std. Predicted Value	-3.359	1.861	.000	1.000	153
Std. Residual	-1.198	3.696	.000	.983	153

a. Dependent Variable: Lp(a) (mg/dL or nmol/L)

Figure S1. Distribution of standardized residuals in the multivariable regression model for Lp(a)

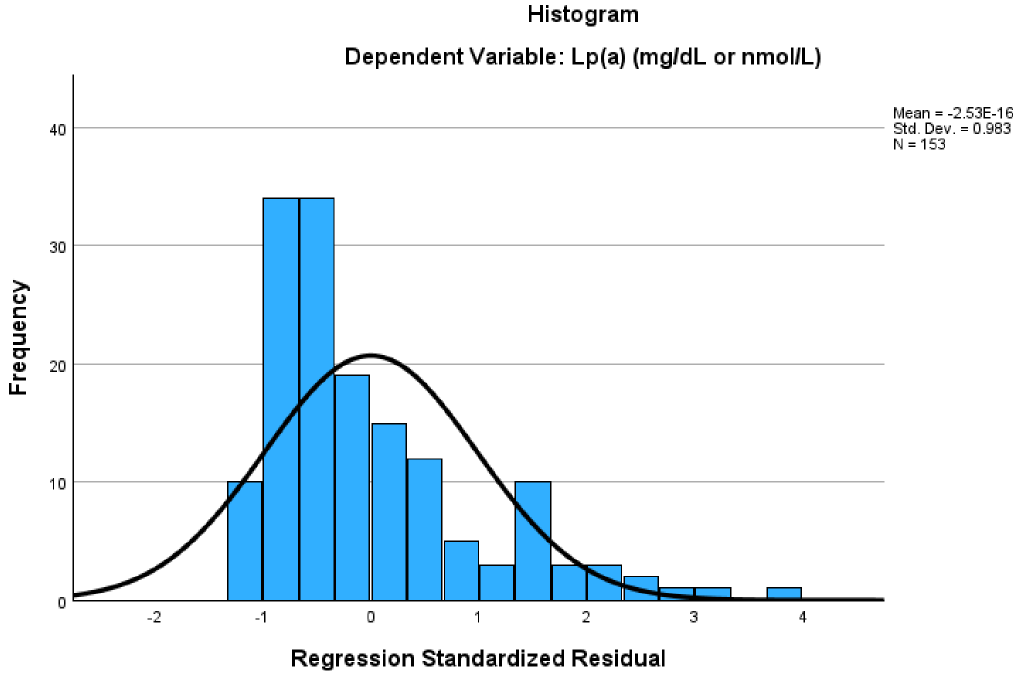

Figure S2. Normal probability plot (P–P) of regression standardized residuals for Lp(a)

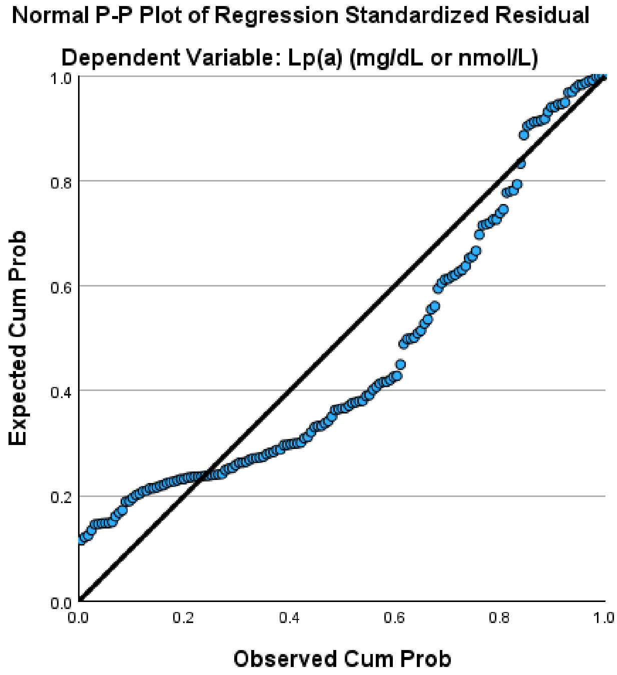

Figure S3. Partial regression plot illustrating the association between LDL-C and Lp(a) after adjustment for covariates

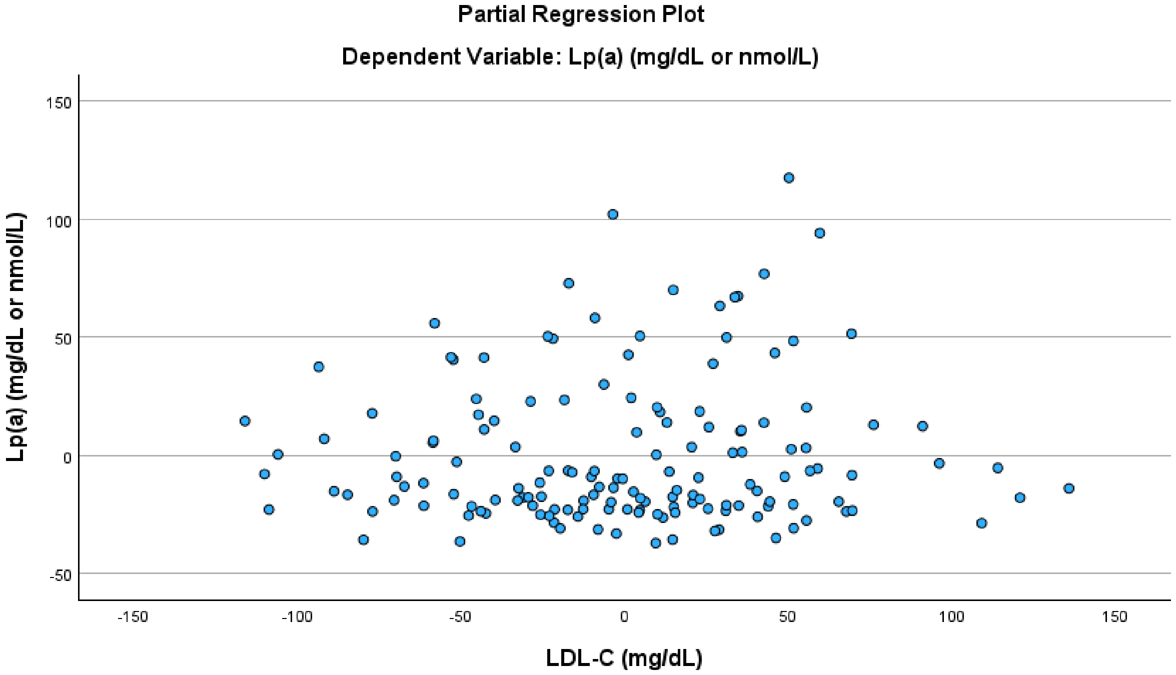

Figure S4. Partial regression plot illustrating the association between triglycerides and Lp(a) after adjustment for covariates

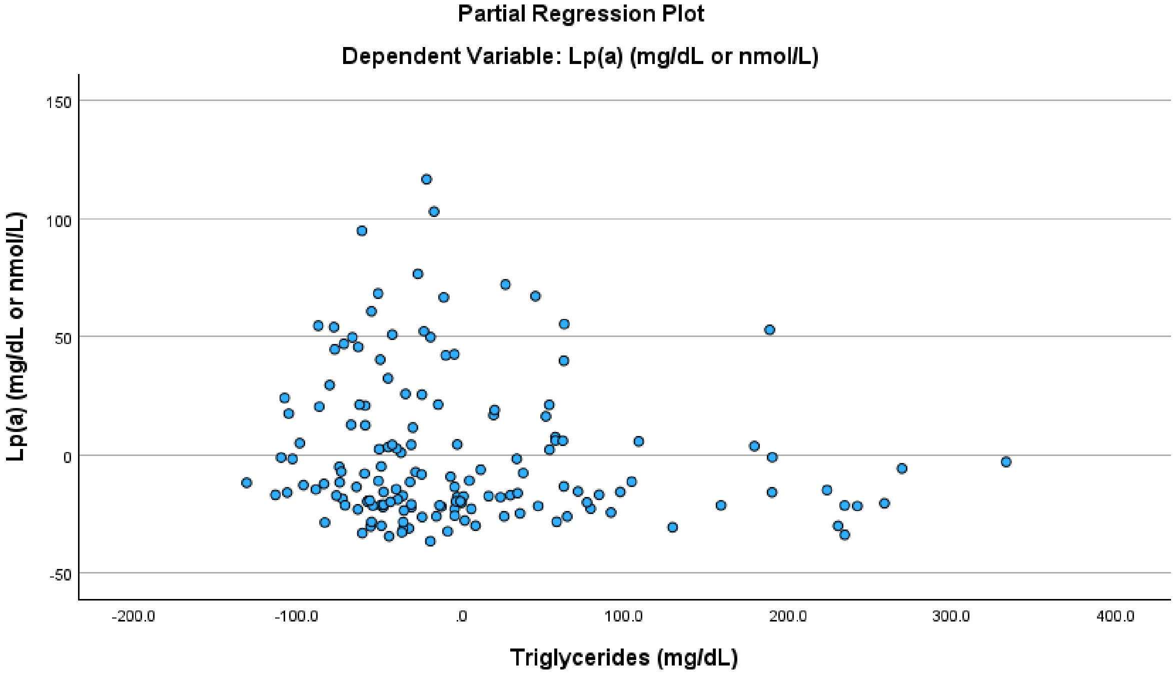

Figure S5. Partial regression plot illustrating the association between BMI and Lp(a) after adjustment for covariates

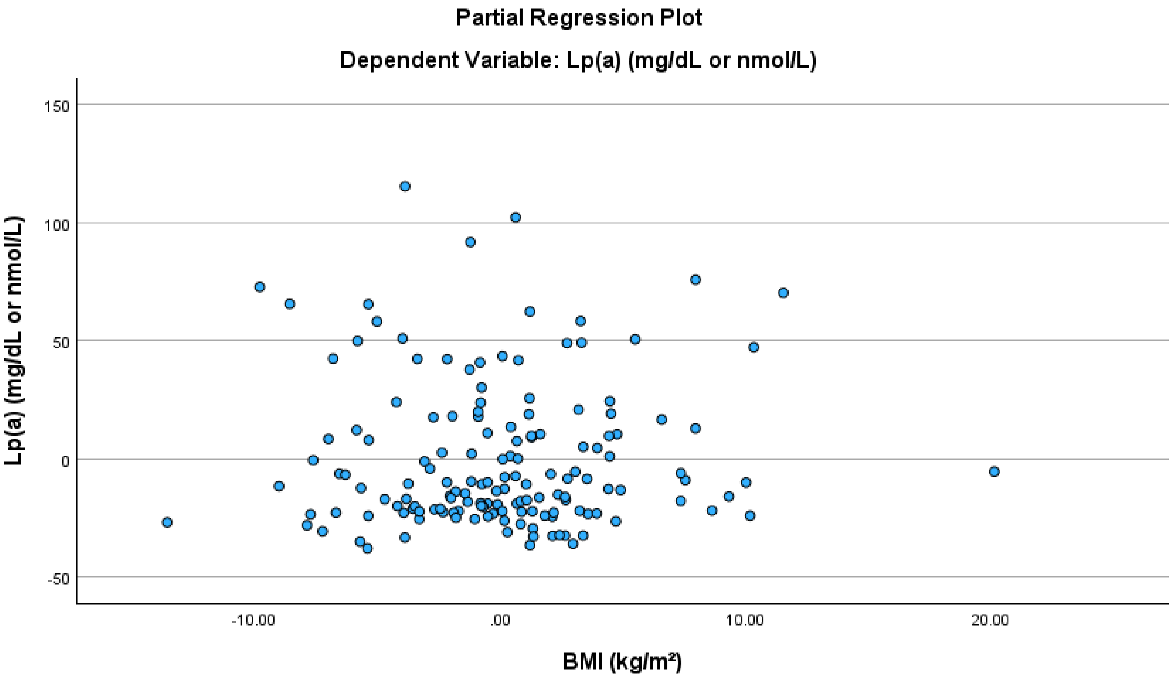

Figure S6. Partial regression plot illustrating the association between Diabetes mellitus and Lp(a) after adjustment for covariates

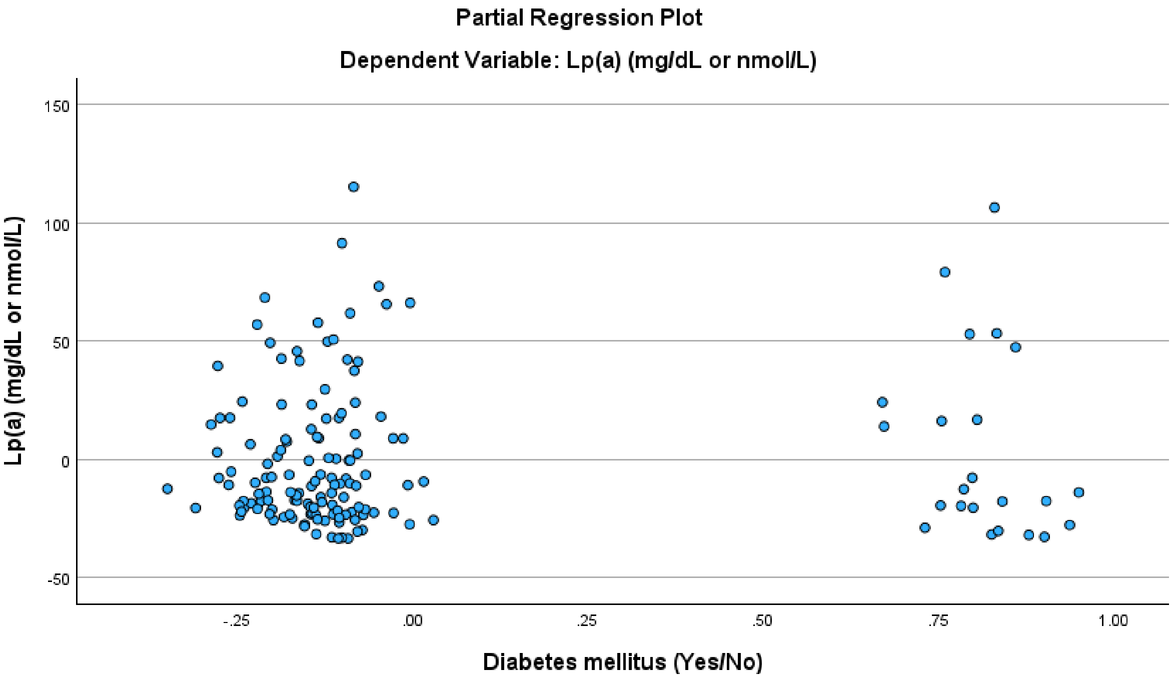

Figure S7. Partial regression plot illustrating the association between male and Lp(a) after adjustment for covariates

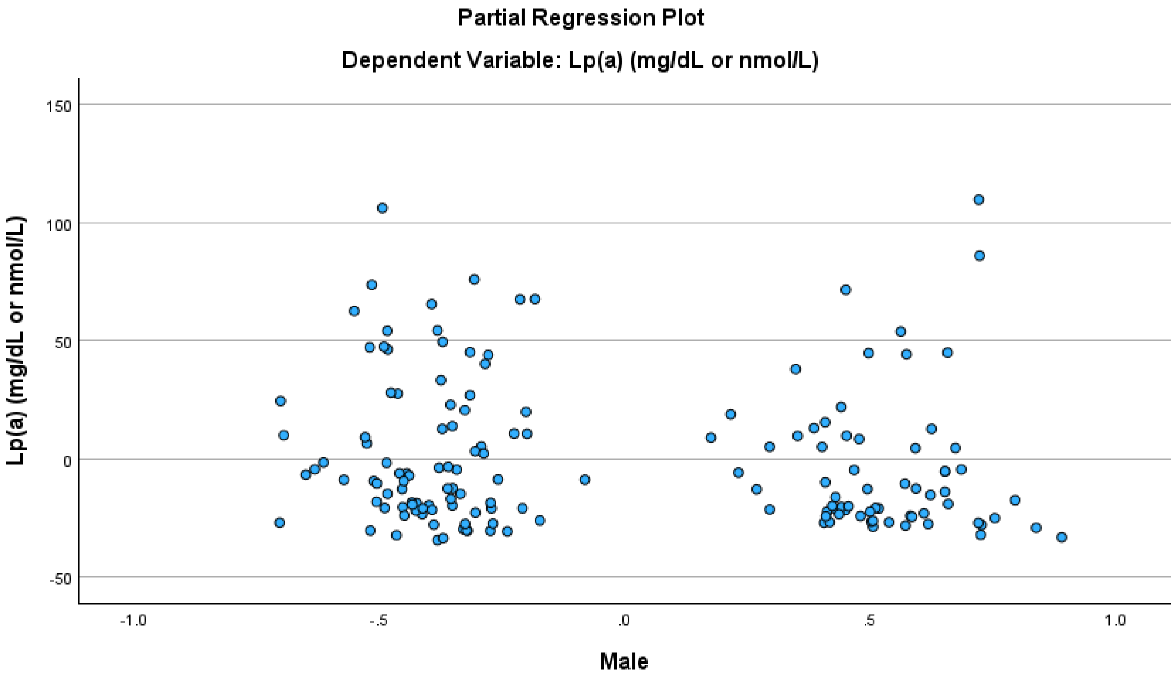

REGRESSION ANALYSIS – HEPATIC AND RENAL INFLUENCE

Table S11. Model specification for multivariable analysis of liver and renal function parameters associated with Lp(a)

Variables Entered/Removed[a]

Model	Variables Entered	Variables Removed	Method
1	Urea (mg/dL), GGT, AST (U/L), Creatinine (mg/dL), ALT (U/L)[b]	.	Enter

a. Dependent Variable: Lp(a) (mg/dL or nmol/L)
b. All requested variables entered.

Table S12. Goodness-of-fit statistics for the regression model with hepatic and renal variables predicting Lp(a)

Model Summary[b]

Model	R	R Square	Adjusted R Square	Std. Error of the Estimate
1	.226[a]	.051	.018	31.295

a. Predictors: (Constant), Urea (mg/dL), GGT, AST (U/L), Creatinine (mg/dL), ALT (U/L)
b. Dependent Variable: Lp(a) (mg/dL or nmol/L)

Table S13. ANOVA results for hepatic and renal predictors of Lp(a)

ANOVA[a]

Model		Sum of Squares	df	Mean Square	F	Sig.
1	Regression	7671.675	5	1534.335	1.567	.173[b]
	Residual	142992.720	146	979.402		
	Total	150664.395	151			

a. Dependent Variable: Lp(a) (mg/dL or nmol/L)
b. Predictors: (Constant), Urea (mg/dL), GGT, AST (U/L), Creatinine (mg/dL), ALT (U/L)

Table S14. Regression coefficients for liver and renal function parameters associated with Lp(a)

Coefficients[a]

Model		Unstandardized Coefficients		Standardized Coefficients		
		B	Std. Error	Beta	t	Sig.
1	(Constant)	56.512	19.996		2.826	.005
	AST (U/L)	.121	.213	.122	.567	.572
	ALT (U/L)	-.124	.177	-.153	-.699	.485
	GGT	-.397	.176	-.201	-2.252	.026
	Creatinine (mg/dL)	7.210	12.954	.048	.557	.579
	Urea (mg/dL)	.194	.246	.065	.791	.430

a. Dependent Variable: Lp(a) (mg/dL or nmol/L)

Table S15. Residual and influence diagnostics for the multivariable regression model including hepatic and renal predictors of Lp(a)

Residuals Statistics[a]

	Minimum	Maximum	Mean	Std. Deviation	N
Predicted Value	9.37	53.75	30.22	7.128	152
Std. Predicted Value	-2.925	3.301	.000	1.000	152
Standard Error of Predicted Value	2.748	25.846	5.637	2.632	152
Adjusted Predicted Value	9.93	80.89	30.53	8.436	152
Residual	-50.752	107.981	.000	30.773	152
Std. Residual	-1.622	3.450	.000	.983	152
Stud. Residual	-1.703	3.494	-.003	1.005	152
Deleted Residual	-77.888	113.233	-.308	32.416	152
Stud. Deleted Residual	-1.714	3.638	.001	1.016	152
Mahal. Distance	.171	102.000	4.967	9.251	152

Cook's Distance	.000	.704	.011	.059	152
Centered Leverage Value	.001	.675	.033	.061	152

a. Dependent Variable: Lp(a) (mg/dL or nmol/L)

Figure S8. Distribution of standardized residuals in the multivariable regression model with hepatic and renal variables

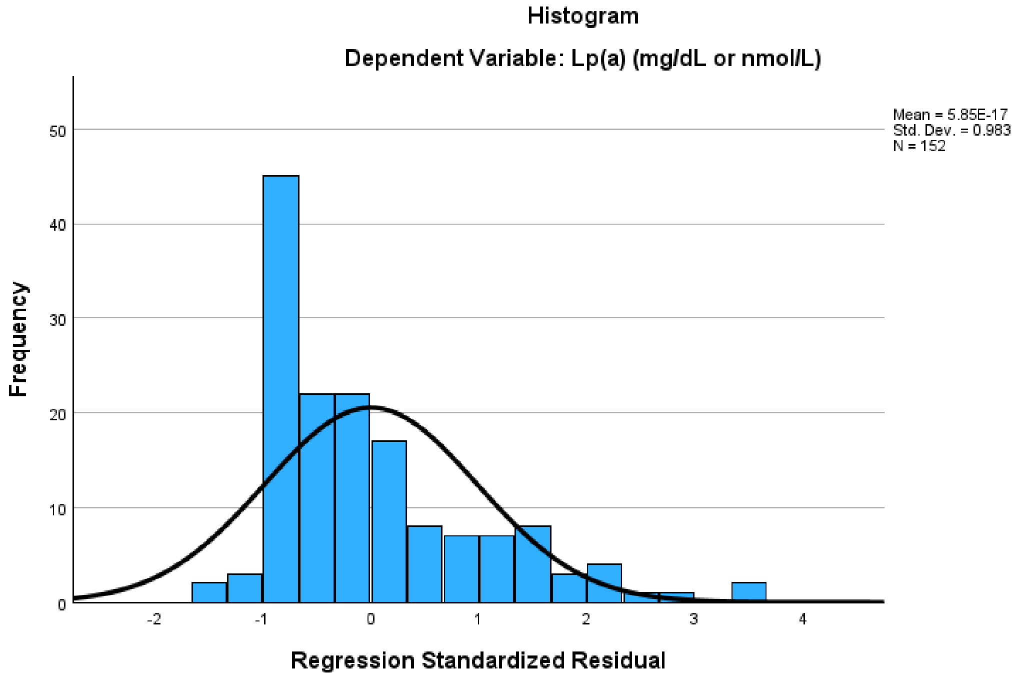

Figure S9. Normal probability plot (P–P) of regression standardized residuals for the hepatic and renal model of Lp(a)

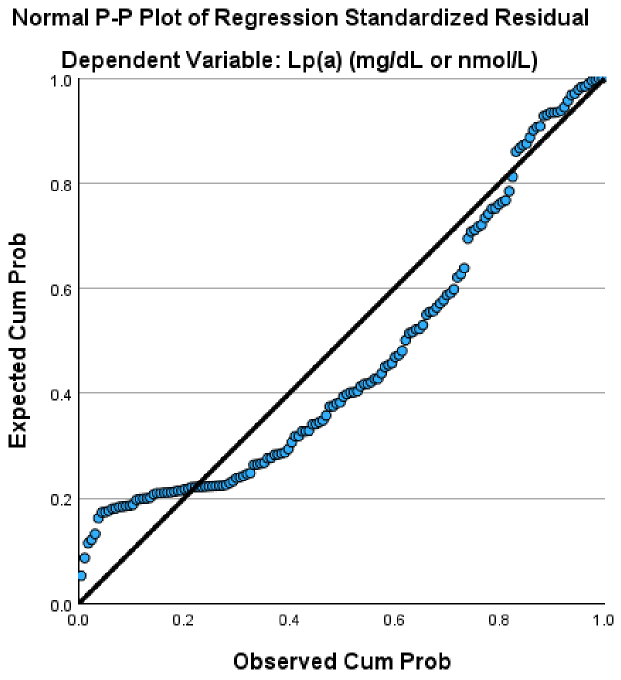

Figure S10. Relationship between observed Lp(a) and standardized predicted values in the regression model

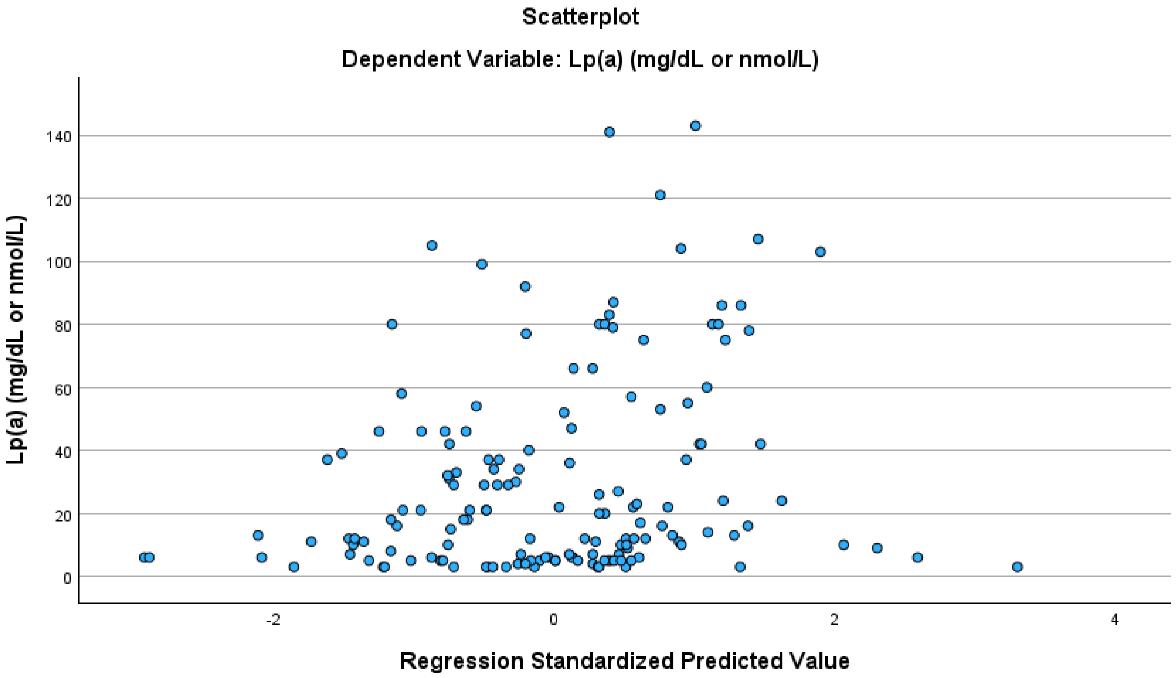

Figure S11. Relationship between Lp(a) values and standardized residuals in the multivariable regression model

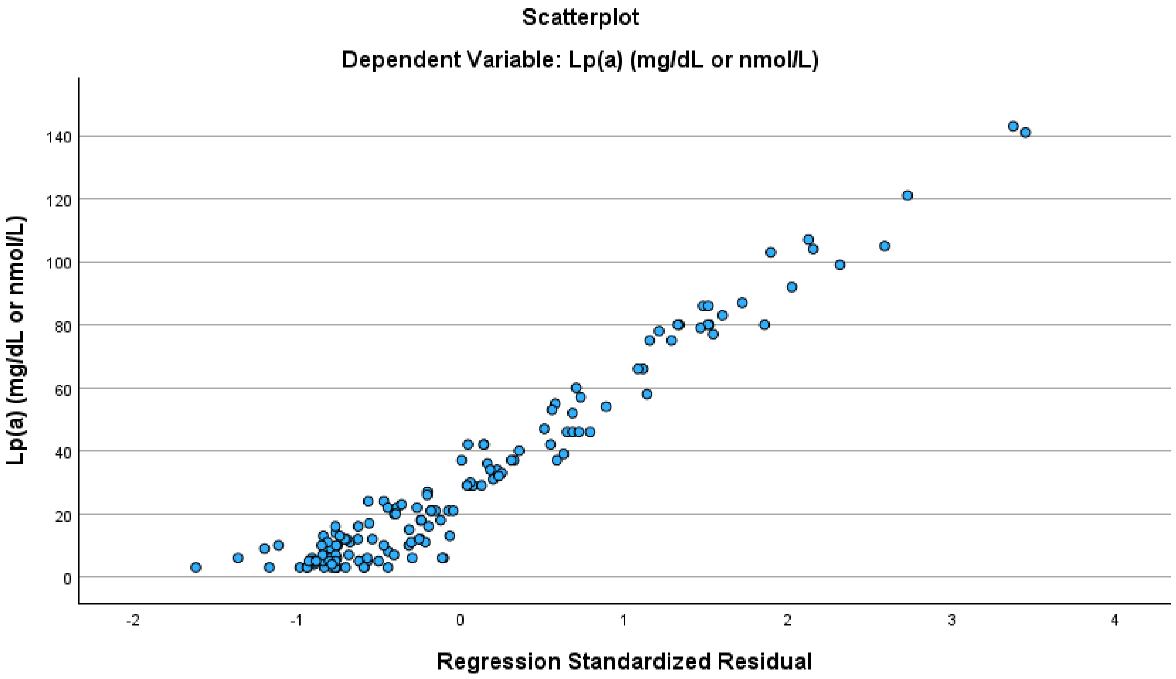

Figure S12. Relationship between Lp(a) values and deleted residuals (PRESS) in the multivariable regression model

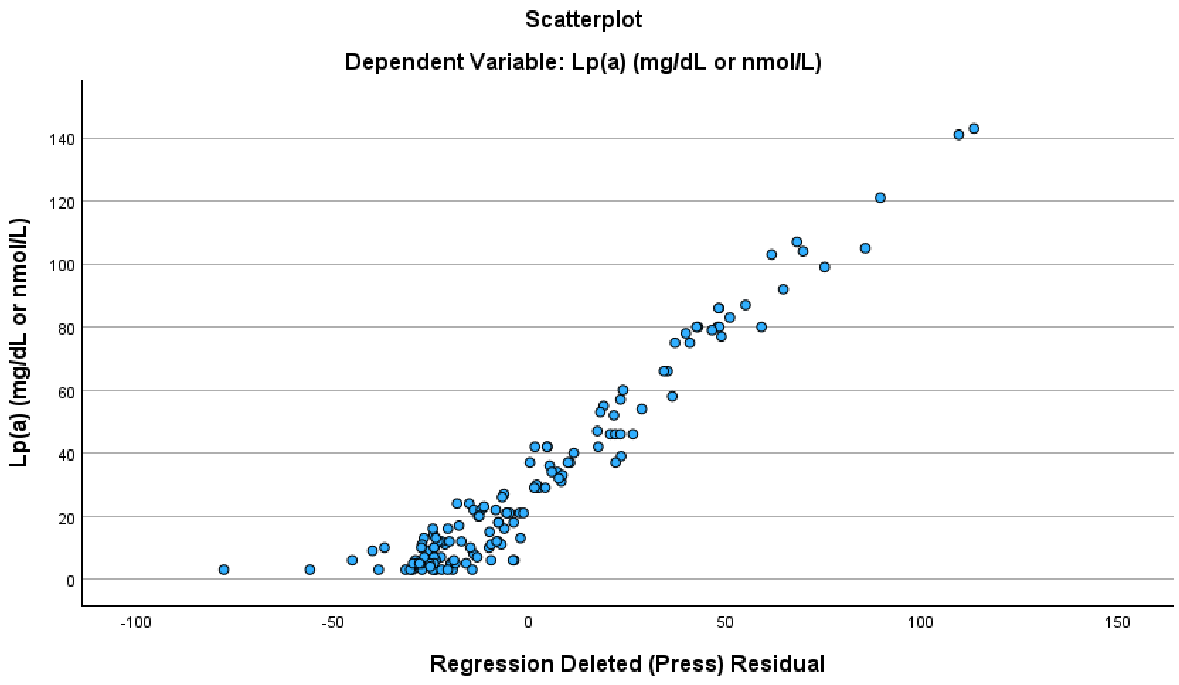

Figure S13. Observed versus adjusted predicted values (PRESS) for Lp(a) in the multivariable regression model

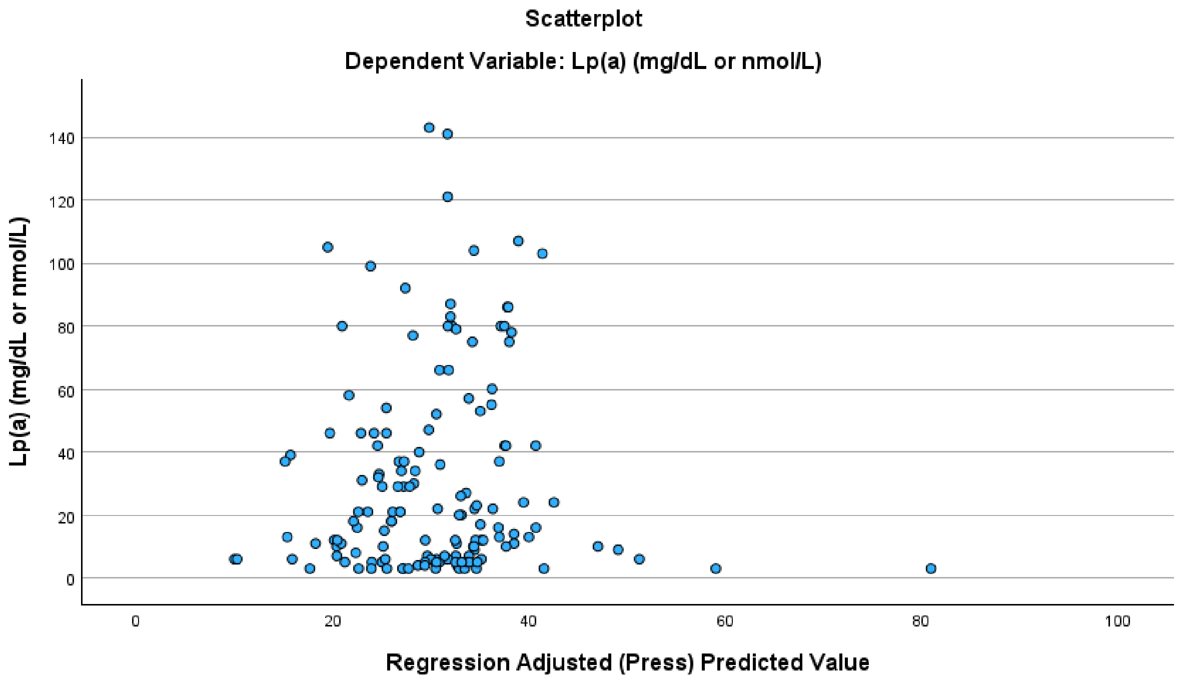

Figure S14. Relationship between Lp(a) values and studentized residuals in the multivariable regression model

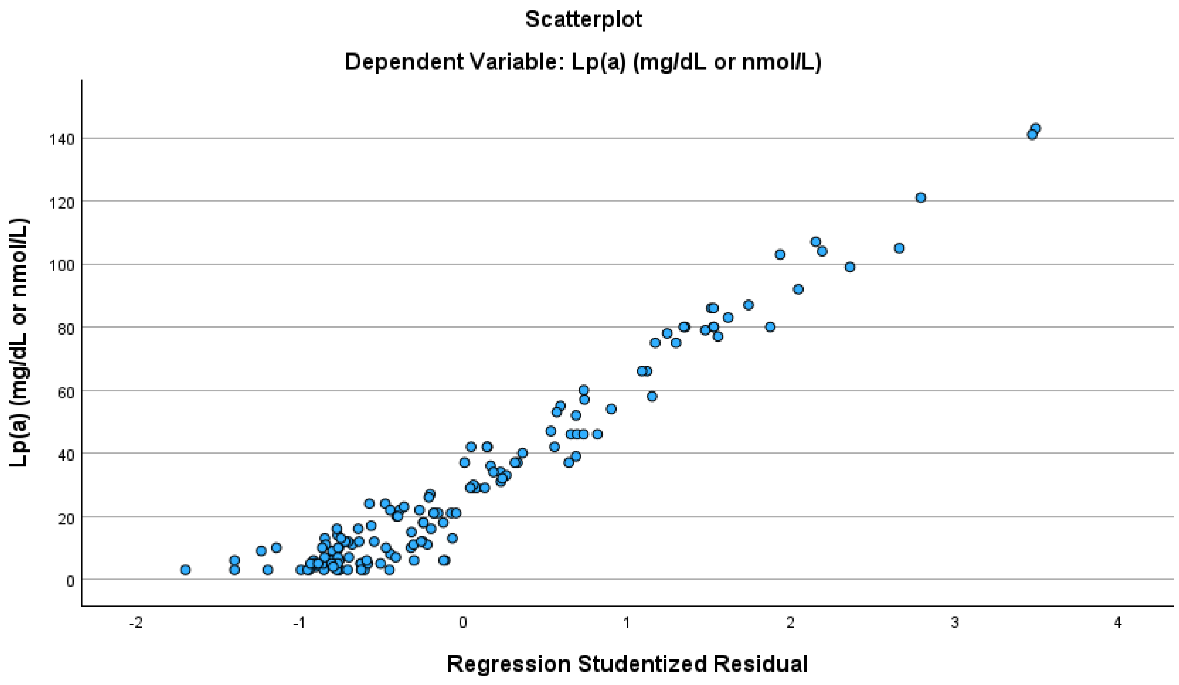

Figure S15. Relationship between Lp(a) values and studentized deleted residuals (PRESS) in the multivariable regression model

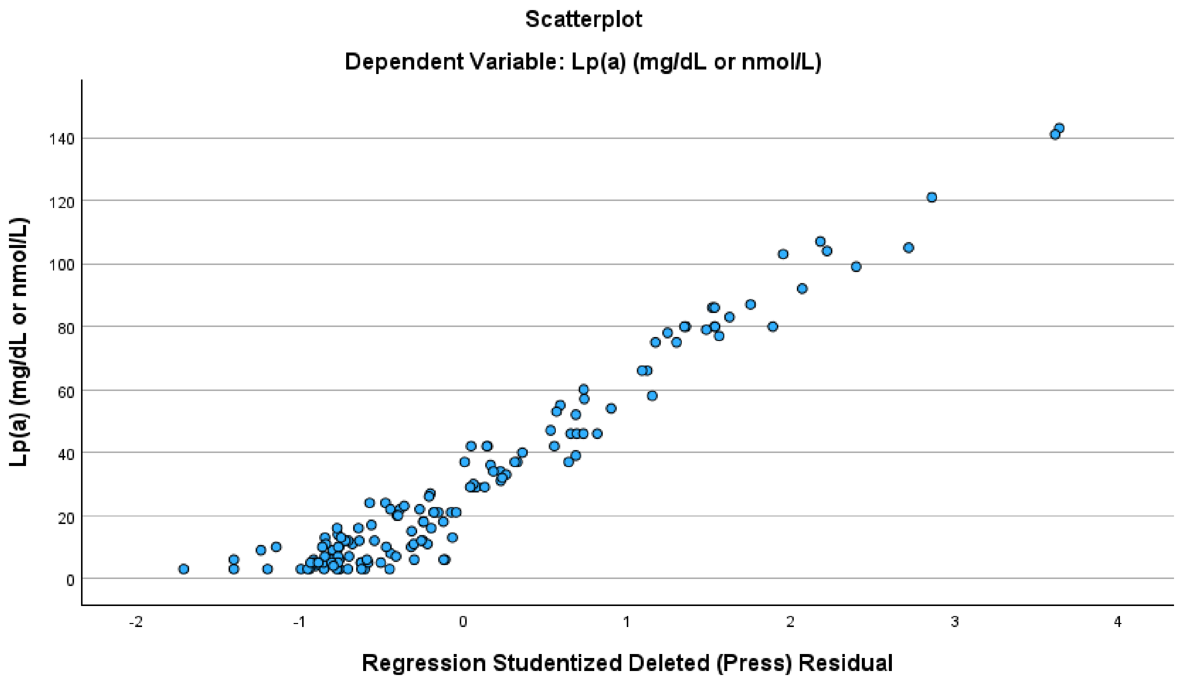

Figure 16. Adjusted relationship between AST and Lp(a) in the multivariable regression model

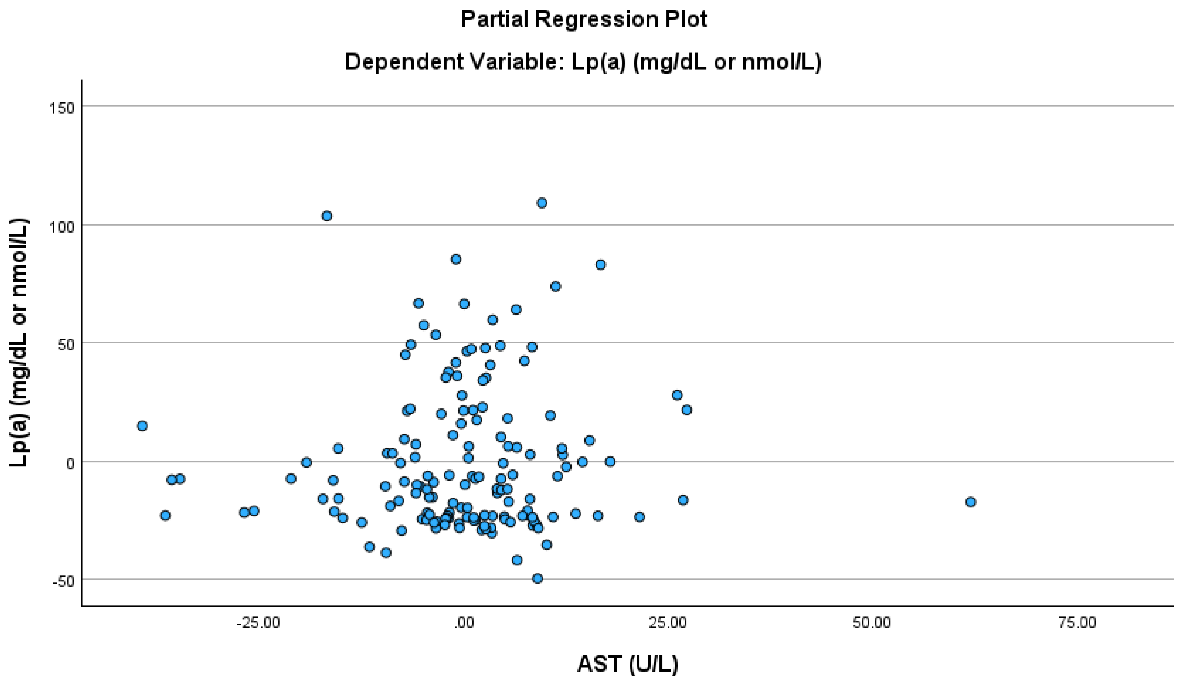

Figure 17. Adjusted relationship between ALT and Lp(a) in the multivariable regression model

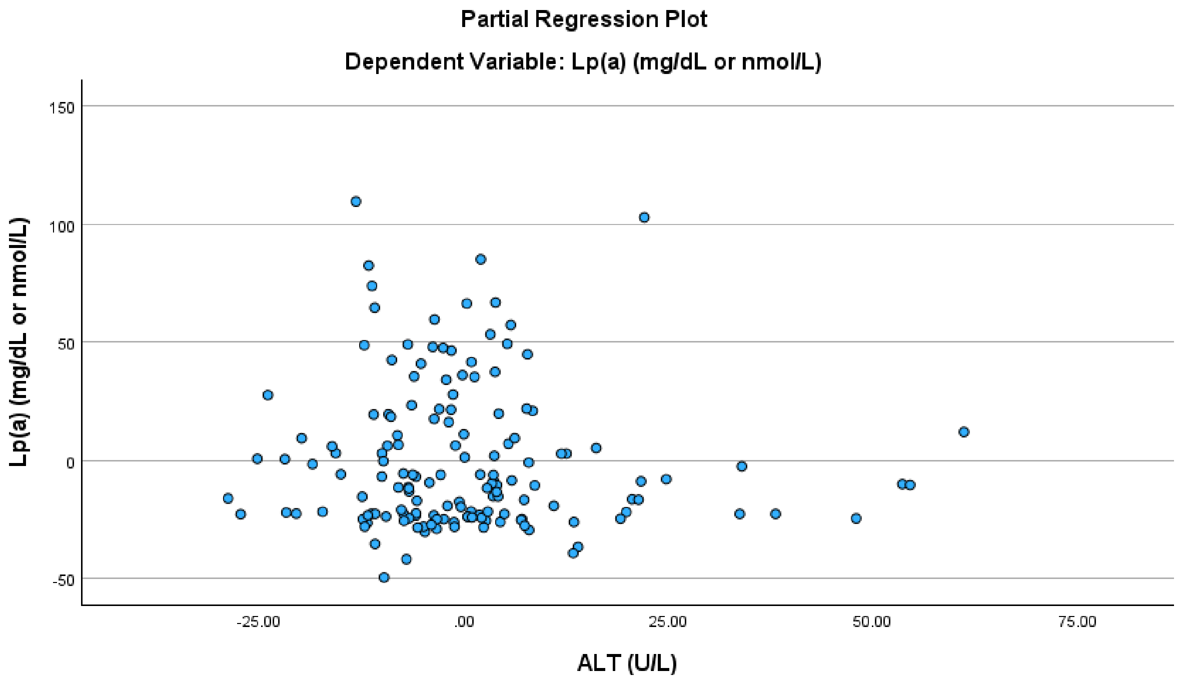

Figure 18. Adjusted relationship between GGT and Lp(a) in the multivariable regression model

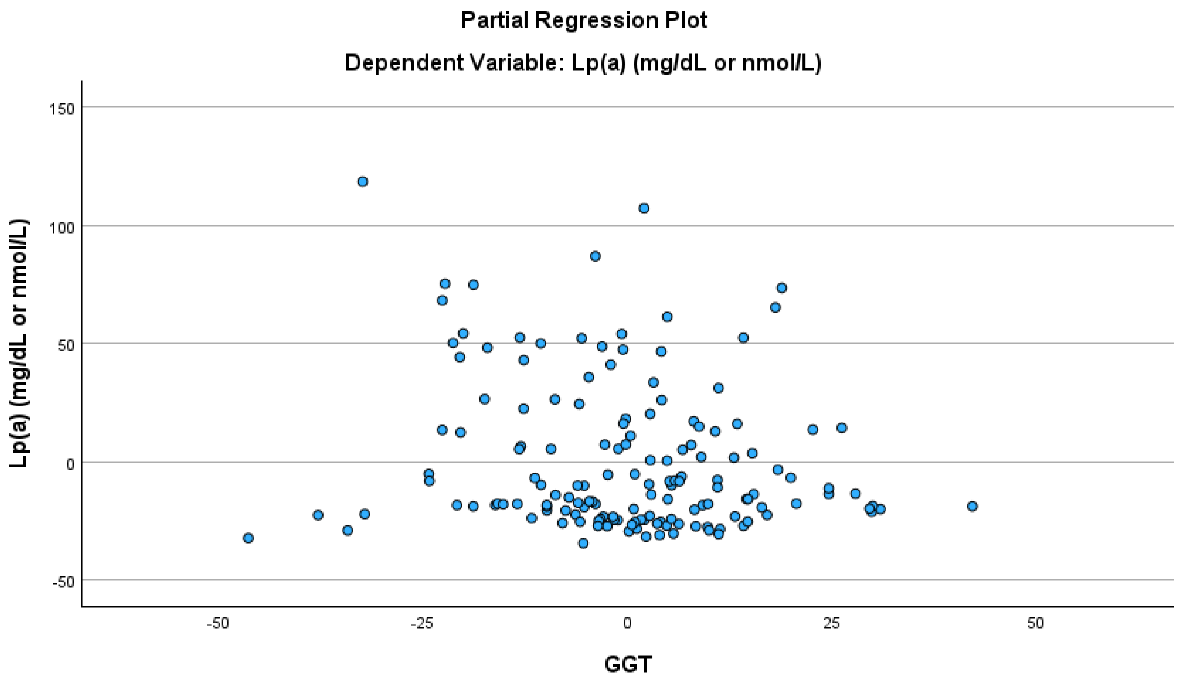

Figure 19. Adjusted relationship between creatinine and Lp(a) in the multivariable regression model

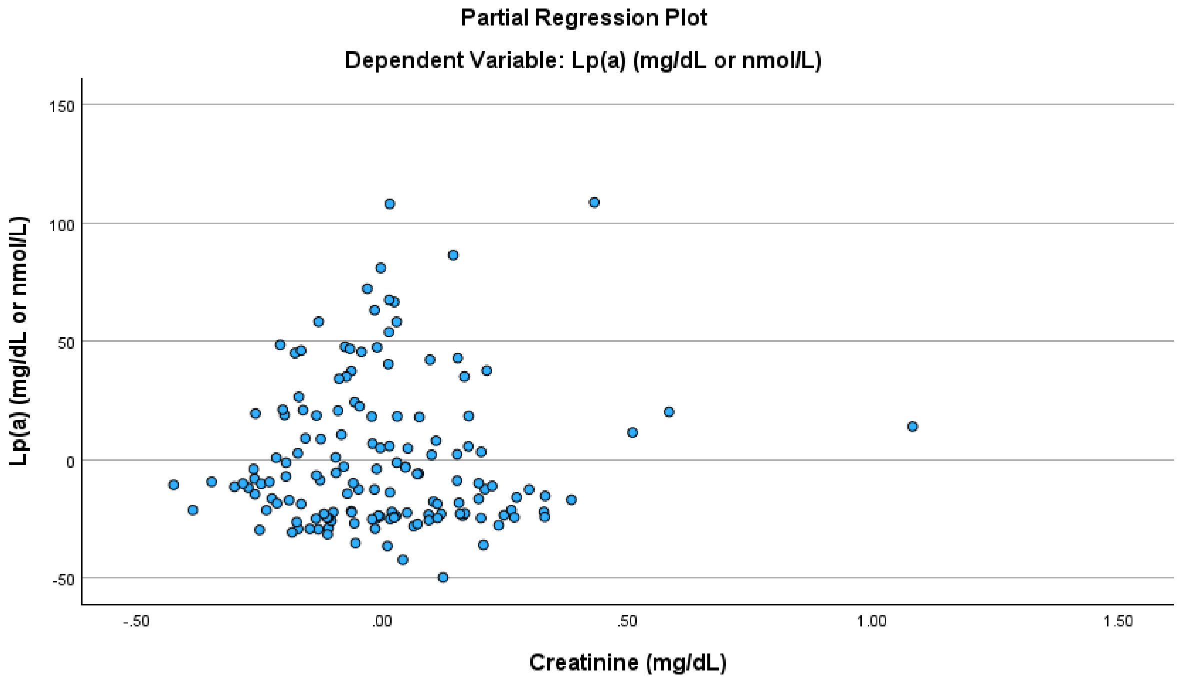

Figure 20. Adjusted relationship between urea and Lp(a) in the multivariable regression model

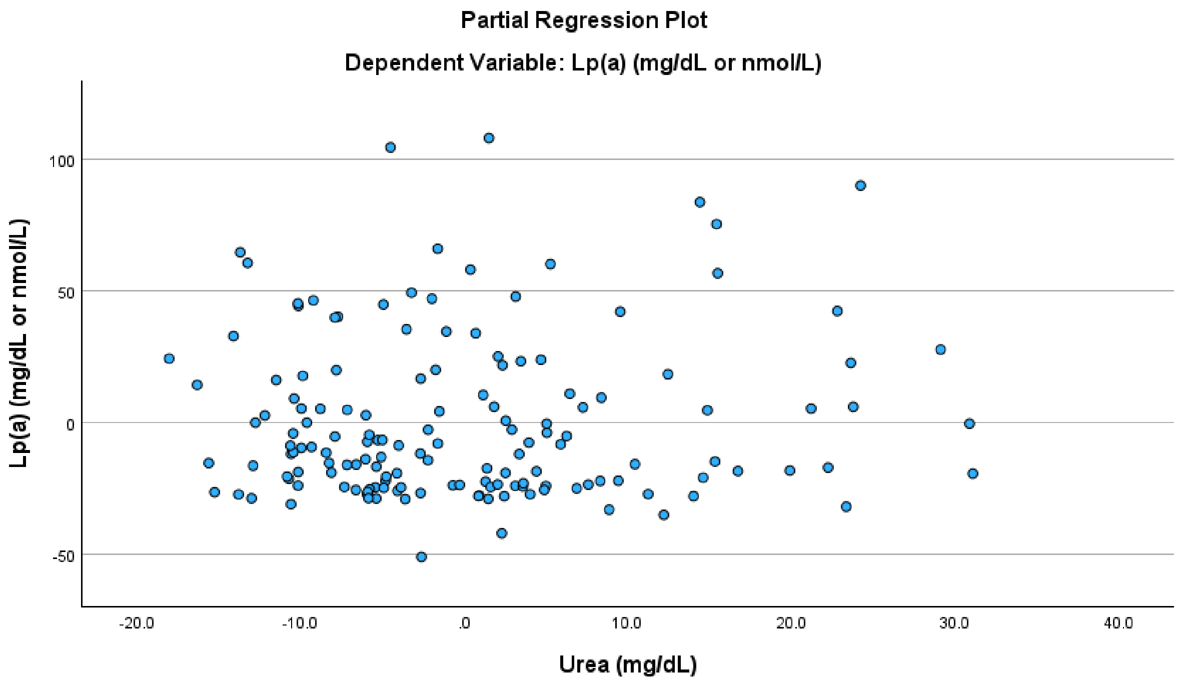

Supplement: Supplementary file 1 [file Datasheet1.pdf]
